# LncRNA BC promotes lung adenocarcinoma progression by modulating IMPAD1 alternative splicing

**DOI:** 10.1002/ctm2.1129

**Published:** 2023-01-17

**Authors:** Qi Wen Chen, Qian Qian Cai, Ying Yang, Shu Dong, Yuan Yuan Liu, Zhong Yi Chen, Chun Lan Kang, Bing Qi, Yi Wei Dong, Wei Wu, Li Ping Zhuang, Ye Hua Shen, Zhi Qiang Meng, Xing Zhong Wu

**Affiliations:** ^1^ Department of Integrative Oncology Fudan University Shanghai Cancer Center Shanghai P. R. China; ^2^ Department of Oncology, Shanghai Medical College Fudan University Shanghai P. R. China; ^3^ Shanghai Key Laboratory of Molecular Imaging Shanghai University of Medicine and Health Sciences Shanghai P. R. China; ^4^ Key Laboratory of Glycoconjugate Research Ministry of Public Health, Department of Biochemistry and Molecular Biology, School of Basic Medical Sciences Fudan University Shanghai P. R. China; ^5^ Department of Pathology Shanghai Pulmonary Hospital, Tongji University School of Medicine Shanghai P. R. China

**Keywords:** alternative splicing, EGFR‐TKI resistance, inositol monophosphatase domain containing 1, lung adenocarcinoma, non‐coding RNA

## Abstract

**Background:**

The therapeutic value of targeted therapies in patients with lung cancer is reduced when tumours acquire secondary resistance after an initial period of successful treatment. However, the molecular events behind the resistance to targeted therapies in lung cancer remain largely unknown.

**Aims:**

To discover the important role and mechanism of lncRNA BC in promoting tumor metastasis and influencing clinical prognosis of LUAD. Materials & Methods: Microarrays were used to screen a comprehensive set of lncRNAs with differential expression profiles in lung cancer cells. The functional role and mechanism of lncRNA were further investigated by gain‐ and loss‐of‐function assays. RNA pull‐down, protein assays, and mass spectrometry were used to identify proteins that interacted with lncRNA. TaqMan PCR was used to measure lncRNA in lung adenocarcinoma and adjacent nontumor tissues from 428 patients. The clinical significance of lncRNA identified was statistically confirmed in this cohort of patients.

**Results:**

In this study, we show that the long non‐coding RNA BC009639 (BC) is involved in acquired resistance to EGFR‐targeted therapies. Among the 235 long non‐coding RNAs that were differentially expressed in lung cancer cell lines, with different metastatic potentials, BC promoted growth, invasion, metastasis, and resistance to EGFR‐tyrosine kinase inhibitors (EGFR‐TKIs), both in vitro and in vivo. BC was highly expressed in 428 patients with lung adenocarcinoma (LUAD) and high BC expression correlated with reduced efficacy of EGFR‐TKI therapy. To uncover the molecular mechanism of BC‐mediated EGFR‐TKI resistance in lung cancer, we screened and identified nucleolin and hnRNPK that interact with BC. BC formed the splicing complex with nucleolin and hnRNPK to facilitate the production of a non‐protein‐coding inositol monophosphatase domain containing 1 (IMPAD1) splice variant, instead of the protein‐coding variant. The BC‐mediated alternative splicing (AS) of IMPAD1 resulted in the induction of the epithelial–mesenchymal transition and resistance to EGFR‐TKI in lung cancer. High BC expression correlated with clinical progress and poor survival among 402 patients with LUAD.

**Disscussion:**

Through alternative splicing, BC boosted the non‐coding IMPAD1‐203 transcript variant while suppressing the IMPAD1‐201 variant. In order to control the processing of pre‐mRNA, BC not only attracted RNA binding proteins (NCL, IGF2BP1) or splicing factors (hnRNPK), but also controlled the formation of the splicing‐regulator complex by creating RNA‐RNA‐duplexes.

**Conclusion:**

Our results reveal an important role for BC in mediating resistance to EGFR‐targeted therapy in LUAD through IMPAD1 AS and in implication for the targeted therapy resistance.

## Graphical abstract

- Abnormally high expression of BC correlates with poor survival of LUAD patients.
- BC is involved in acquired resistance to EGFR‐targeted therapies.
- BC restores the epithelial–mesenchymal transition suppressed by IMPAD1.
- BC promotes IMPAD1 alternative splicing via an interaction with hnRNPK and NCL.

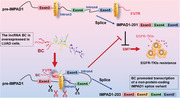

## 1 INTRODUCTION

Metastasis is the leading cause of death in patients with lung cancer.^1^ Of all lung cancers non‐small cell lung cancer (NSCLC) accounts for ∼85%.^2^ The poor prognosis of patients with NSCLC is usually caused by remote metastasis, resulting in disease progression and treatment failure.^3^, ^4^ Two classes of molecularly targeted therapies, EGFR‐tyrosine kinase inhibitors (TKIs) and anaplastic lymphoma kinase inhibitors, were recently introduced into clinical practice to treat patients with NSCLC.^5^ Since then, great progress has been made using several EGFR‐TKIs in the treatment of advanced NSCLC. Gefitinib is an EGFR‐TKI that is highly effective in patients with EGFR‐mutated NSCLC.^6^ Afatinib is an irreversible EGFR/HER2 inhibitor used to treat NSCLC.^7^ Osimertinib is an irreversible EGFR‐TKI used to treat T790M‐mediated lung cancer that is resistant to other EGFR inhibitors.^8^ In the clinical application of EGFR‐TKIs (first or second generation), about 70% of patients experience a decrease in sensitivity to the drugs after 6–14 months of treatment, which ultimately affects the clinical efficacy of the drugs.^9^, ^10^ Some acquired resistance to EGFR‐TKIs is related to a secondary point mutation in codon 790 of exon 20 (T790M) of the EGFR gene,^11^ and some may involve Met amplification,^12^ HER2 amplification,^13^ or the epithelial–mesenchymal transition (EMT),^14^ whereas other resistance mechanisms are still unclear.^15^

Long non‐coding RNAs (lncRNAs) are defined as transcripts longer than 200 nucleotides that have no protein‐coding potential.^16^ Although almost all lncRNAs contain short reading frames, because none of the reading frames are >100 codons; they are defined as non‐coding or have strong coding potential signals. It has become clear, however, that lncRNAs have important roles in biological and pathological processes.^17^ There is evidence that lncRNA deregulation contributes to a variety of diseases, including cancer.^18^, ^19^, ^20^ A number of lncRNAs were found to be aberrantly expressed in a broad spectrum of cancers and to play roles in various aspects of tumorigenesis.^21^ Little is known, however, about what roles lncRNAs play in NSCLC resistance to EGFR‐TKIs. In this study, we show that the expression of the lncRNA BC009639 (BC) promotes lung cancer growth and metastasis, and resistance to EGFR‐TKI targeted therapies by regulating EMT.

## 2 METHODS

### 2.1 LncRNA microarray expression profiling

Microarray profiling was conducted in the laboratory of Aksomics Inc. (Shanghai, China). The microarray was analysed using the nrStar Functional LncRNA PCR chip software, version 1.0 (Arraystar, Rockville, USA). The hierarchical clustering analysis was carried out using the TBtools, a platform‐independent software.^22^

### 2.2 Patients and tissue microarray

Tumour tissues and adjacent non‐tumour tissues were collected from patients (*n* = 40 + 428) at Fudan University Shanghai Cancer Center, Shanghai Chest Hospital, Shanghai Pulmonary Hospital and Affiliated Hospital of Guilin Medical University, Wenzhou Medical University.^23^ All patients were pathologically confirmed to have lung cancer. Histopathological grading was performed according to the criteria of Hoffman PC.^24^

### 2.3 Cell culture and treatment

BEAS‐2B bronchial epithelial cells were cultured in Gibco LHC basal medium supplemented with 10% foetal bovine serum. PG‐LH7, PG‐BE1, 95C, 95D, the H460 human giant‐cell lung carcinoma cells and the A549 NSCLC cells were from Cell Bank of Type Culture Collection of Shanghai Institute of Biochemistry & Cell Biology, Chinese Academy of Science and cultured in Gibco RPMI‐1640 medium (Invitrogen, Carlsbad, CA) supplemented with 10% FBS. Cells were treated with the selective EGFR inhibitors osimertinib, gefitinib, afatinib; the EGFR‐TKI erlotinib; the multikinase inhibitors sunitinib and sorafenib; the mTOR inhibitor everolimus; and the MEK1/2 inhibitor trametinib (all from Meilun Biotechnology Co., Ltd, Dalian, China).

### 2.4 Plasmid construction and transfection

BC wild‐type cDNA and mutant cDNAs of different lengths were cloned into pcDNA3.1b. However, the CDS sequence of inositol monophosphatase domain containing 1 (IMPAD1) (CCDS:CCDS6169.1) was also cloned into pcDNA3.1b. The shRNAs targeting human IMPAD1 and hnRNPK were constructed into the pSilencer 4.1. The BC knockout was mediated by the CRISPR‐Cas9 system (Inovogen Tech.Co), whereas the guide RNA were designed to use the CRISPR Design (http://crispr.mit.edu/) (Table S1). Cells were transfected with the vectors or siRNA targeting human NCL using Lipofectamine 2000 (Invitrogen) according to the manufacturer's instructions.

### 2.5 Separation of nuclear and cytoplasmic fractions

The nuclear and cytoplasmic fractions of cells were extracted using a Nuclear and Cytoplasmic Protein Extraction Kit (Beyotime P0028) containing 2 U/μl RNase Inhibitor (Beyotime R0102). Cells were washed three times with PBS on ice followed by centrifugation at 300 × *g* for 5 min. Cell pellets were resuspended in cytoplasmic extraction buffer from the Extraction kit, incubated on ice for 10 min and then centrifuged at 500 × *g* for 5 min at 4°C. Collect the cytoplasmic fraction in the supernatant, and the nuclear pellets were homogenized with the nuclear disruption buffer from the Extraction kit.

### 2.6 RNA extraction and RT‐qPCR

Total RNA, cytoplasmic RNA and nuclear RNA were extracted using TRIZOL reagent (Invitrogen) and reverse transcribed to cDNA. β‐Actin was used as an internal control. Real‐time PCR was performed using a Bio‐Rad CFX Connect Real‐Time PCR system (Bio‐Rad). Relative expression levels were calculated as ratios normalized against β‐actin. The PCR primer sequences are listed in Table S1.

### 2.7 Western blot

Proteins were separated by 8% SDS–PAGE and transferred to a PVDF membrane. The membranes were blocked and incubated first with primary antibodies at 4°C overnight and then with horseradish peroxidase–conjugated secondary antibody. Antibodies against NCL, hnRNPD, hnRNPC, hnRNPK, SF3B4, IGF2BP1, RBM3, MGMT, hnRNPUL1, polyadenylate‐binding protein 1 (PABPC1), ZEB‐1 and AGO2 were obtained from Proteintech (Wuhan, China). Antibodies against GAPDH, AKT, phospho‐AKT (T308), phospho‐AKT (Y326) and phospho‐AKT (S473) were obtained from Bioworld Technology, Inc. (MN, USA). Antibody against IMPAD1 was obtained from Abcam (Cambridge, UK). Antibody against hnRNPCL1 was obtained from Abgent (Taiwan, China). Antibodies against E‐cadherin, Src and U1 SnRNP70 were obtained from Santa Cruz (CA, USA). Antibodies against N‐cadherin, vimentin, Snail, and Slug were obtained from GeneTex (CA, USA). Antibodies against phospho‐Src (T416), PDPK1, phospho‐PDPK1 (S241), mTOR, phosphor‐mTOR (S2448) EGFR, Phospho‐EGFR (Tyr1173), VEGFR and Phospho‐VEGFR (Tyr951) were obtained from Cell Signaling Technology (Danvers, MA, USA). The densities of developed protein bands were analysed using TotalLab v2.01 (Nonlinear Dynamics Ltd).

### 2.8 Colony formation assay

Colony formation was measured using an assay described in our previous report.^25^

### 2.9 Cell cycle analysis

Cells were harvested, washed and fixed in pre‐cooled 75% ethanol mixed with PBS at −20°C overnight. The cells were then washed twice with pre‐cooled PBS, resuspended in 500 μl pre‐cooled PBS, including 100 μg/ml RNase (Thermo Scientific, USA), and incubated at 37°C for 30 min before staining. Then, 50 μg/ml propidium iodide was added, and the cells were incubated at room temperature in the dark for 15 min. All cell samples were harvested and analysed on a flow cytometer FACS scan (Cytomics FC 500, Beckman Coulter).

### 2.10 Migration, invasion and wound healing assays

For migration assays, cells were transfected and placed in the upper chamber of the Millicell insert (8 μm pore size; Millipore, Billerica, MA) in serum‐free medium. Whereas in the invasion assay, coated the upper side of the bottom membrane of the Millicell insert with a 1:8 dilution of 50 mg/L Matrigel (BD, USA). Medium containing 10% FBS was added to the lower chamber. After incubation, cells that migrated through the membrane were fixed, stained and counted. Wound healing assays were performed as previously reported.^26^

### 2.11 MTT assay

Cell viability was measured by MTT assay. A549 cells 95D or PC9 cells were plated at a concentration of 1 × 10^4^ cells/well on 96‐well culture plates at 37°C. After treatment or transfection, 10 μl MTT work solution was added, and the cells were incubated for another 2 h. Then, 100 μl dimethyl sulfoxide was added, and absorbance at 570 nm was measured using an MQuant Universal Microplate Spectrophotometer (BioTek Instruments, USA).

### 2.12 In situ hybridization and fluorescent in situ hybridization (FISH)

In situ hybridization and fluorescence in situ hybridization (FISH) assays to detect BC were conducted with an ISH Detection Kit II and ISH Detection Kit V (Boster, CHINA) according to the manufacturer's protocol. The probe GCGGTCTAGAACTAGTCGTCCCACAGAAGAGACAGTGCTCTGCCATGATGACAGG‐biotin was designed by our laboratory and synthesized by Sangon Bioengineering (Shanghai) Co., Ltd. The probe was used at a concentration of .5–2 μg/ml on a tissue array or on 4% paraformaldehyde‐fixed slides with monolayers of 95C or 95D cells. In situ hybridization was performed at 37°C overnight, followed by visualization using SABC‐AP and BCIP/NBT, whereas FISH used SABC‐FITC for visualization. The slides were then observed under a microscope (Olympus, Tokyo, Japan).

### 2.13 Metastasis model

The male nude mice aged 4–6 weeks were purchased from Charles River, China. A549 cells were stably transfected with BC or non‐coding vector with G418 resistance. Transfected cells (1 × 10^6^) in 100 μl PBS were injected into the tail veins of BALB/C nude mice. The mice were sacrificed and dissected 4 weeks after injection. Death rates prior to sacrifice and metastatic foci in the lungs and pulmonary vessels were counted. All animal studies were conducted under approved guidelines of the Animal Care and Use Committee of Fudan University.

### 2.14 RNA pull‐down and mass spectrometry

RNA pull‐down analysis was performed as previously described.^27^ In brief, BC was transcribed in vitro using T7 RNA polymerase, labelled with biotin (Roche, Mannheim, Germany) and then incubated with cell protein extracts, which were then conjugated with streptavidin beads (Vector Laboratories, CA, USA) and washed. The bead‐associated proteins were resolved by SDS‐PAGE, and then the stained protein bands were cut from the gel and transferred into 1.5 ml EP tubes. After centrifugation and drying for 15 min, 10 μl trypsin (20 ng/μl, dissolved in 25 mmol/L NH4HCO3) was added and digested at 37°C overnight. The lysate were dissolved in 2%ACN/98%H_2_O/.1%FA solution and analysed by LC‐LTQ‐MS (TripleTOF 5600, AB Sciex, Framingham, MA). In addition, the spray voltage was 2.2 kV, whereas the temperature of capillary electrophoresis was 200°C. Data were analysed using ProteinPilot software V4.4 (AB Sciex, Framingham, MA).

### 2.15 Protein microarrays

BC RNA transcription driven by the T7 promoter was performed in vitro using the MEGAscript T7 Kit (Ambion, USA). The RNA was labelled with the fluorescent dye Cy5 according to the manufacturer's instructions. The HuProt 20K Human Protein Microarray (CDI Lab, Mayaguez, USA) was used for BC analysis.

### 2.16 RNA immunoprecipitation (RIP)

RNA immunoprecipitation (RIP) was performed using the Magna RIP Kit (Millipore, USA) according to the manufacturer's instructions. Briefly, after cells were lysed in RIP lysis buffer, 100 μl whole‐cell extract was incubated with RIP buffer containing magnetic beads conjugated with the target antibody. Normal mouse IgG was used as a negative control. The samples were then incubated with proteinase K and shaken to digest the protein. Then, immunoprecipitated RNAs were purified, and qRT‐PCR was performed to detect BC or IMPAD1 mRNA.

### 2.17 PAR‐CLIP data analysis

PAR‐CLIP DNA sequencing reads were downloaded from the NCBI GEO database (GSE94781 (hnRNPC PAR‐CLIP), GES91621 (hnRNPK PAR‐CLIP), GES52971 (hnRNPD PAR‐CLIP), GES91619 (SF3B4 PAR‐CLIP), GSM994703 (RBM3 PAR‐CLIP) and GSE92200 (IGF2BP1 PAR‐CLIP)). After the removal of adapter sequences, the sequencing reads were aligned to the human genome and transcriptome (GRCh37/hg19) using IGV (v2.4.8, http://www.igv.org/). Uniquely mapped reads were overlapped to define binding clusters. For each cluster, the crosslinking position was defined as the position with the highest number of T to C conversions.

### 2.18 RNA sequencing (RNA‐Seq)

We overexpressed BC in both 95C and 95D cells, the total RNA of each sample was isolated by TRIzol, and mRNA was enriched by Dynabeads Oligo (dT) 25 beads (Invitrogen). Three replicates of RNA sequencing (RNA‐Seq) libraries for each sample were constructed using the KAPA Stranded RNA‐Seq Library Prep Kit (Illumina) according to the manufacturer's instructions. The constructed library was identified by Agilent 2100 Bioanalyzer and quantified by qPCR. The pooled different sample libraries were sequenced using an Illumina NovaSeq 6000 sequencer. We mapped all of the RNA‐Seq data to the GRCh37.p13 genome from GENCODE using HISAT2 (version 2.1.0).

### 2.19 Gene set enrichment analysis (GSEA)

The gene set enrichment analysis (GSEA) software (version 4.0.3, www.broadinstitute.org/gsea/) was used to identify gene signatures of samples with high or low BC (ROC cut‐off = 4.1235) expression or in lung adenocarcinoma (LUAD) compared with non‐tumour from the TCGA dataset. Pearson's correlation coefficient was used as the metric to rank genes in terms of differential expression between samples. In addition, when we performed GSEA on RNA‐Seq data of BC‐overexpressed 95C and 95D cells, Diff_of_Classes was used as the metric to rank genes in terms of differential expression between BC overexpression and Mock phenotype. The results of the GSEA were expressed using normalized enrichment scores that take into account the sizes and correlations between gene sets and the expression dataset (*p* < .01).

### 2.20 Statistical analysis

All experimental data were presented as the mean ± SEM of at least three independent experiments. Graphs were prepared using GraphPad Prism software v8 (San Diego, CA). Statistical differences were measured using one‐way ANOVA and Student's *t*‐test. Pearson correlation analysis was used for analyses of linear correlation. Statistical analysis was performed using SPSS software v22 (IBM). *p*‐Values <.05 were considered significant.

## 3 RESULTS

### 3.1 The lncRNA BC is overexpressed in lung adenocarcinoma (LUAD) cells

**FIGURE 1:**
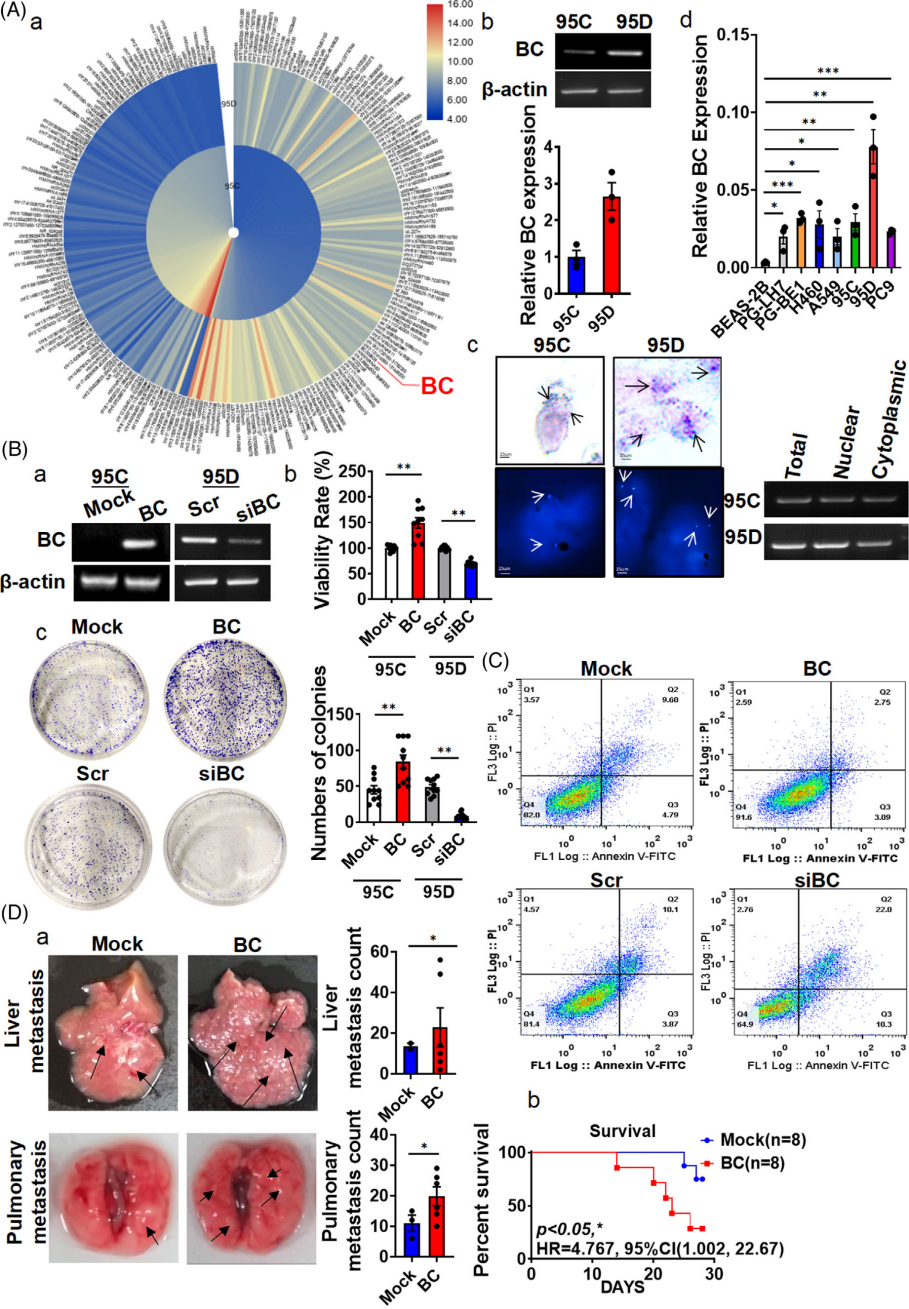
BC overexpressed in lung adenocarcinoma (LUAD) cells, promoted lung cancer metastasis. (A) (a) Hierarchical clustering of 235 differentially expressed long non‐coding RNAs (lncRNAs) (|fold change| > 3) was performed using microarray data. Red, high expression; blue, low expression. (b) The expression of BC in 95C and 95D cells was analysed by RT‐PCR. (c) BC in nuclear and cytoplasmic fractions were detected by in situ hybridization, fluorescence in situ hybridization (left) and RT‐PCR (right). Arrows indicate positive hybridization sites. (d) BC expression was measured by RT‐qPCR in BEAS‐2B, PG‐LH7, PG‐BE1, H460, A549, PC9, 95C and 95D cells. (B) (a) Overexpression and knock‐down of BC were validated by RT‐PCR. (b) The viability of 95D cells was analysed by the MTT assay after BC overexpression or knock‐down. (c) Colony formation assays were performed with BC‐overexpressing and BC‐silenced cells. Quantitative analysis is shown on the right. (C) Flow cytometry was performed to measure cell cycle progression after ectopic expression or silencing of BC. (D) (a) Representative images of livers and lungs of nude mice, 4 weeks after tail‐vein injection (*n* = 8). Arrows indicate metastatic foci. Liver and pulmonary metastasis foci were quantitatively analysed. (b) The death rate of both groups is summarized. Data are represented as mean ± SEM of at least three independent experiments. *p < .05, **p < .01

To screen for lncRNAs associated with lung cancer metastasis, we employed a pair of lung cancer cell lines, 95D and 95C, which have the same genetic background but different metastatic potentials. Microarray expression analysis identified 235 lncRNAs that were differentially expressed between the two cell lines (fold change >3, *p* < .05; Figure 1A‐a, Table S2). Among them, BC (BC009639, ch8:57870492‐57871354, GRCh37/hg19) was one of the most highly upregulated lncRNAs in the metastatic 95D cells in comparison with the nonmetastatic 95C cells, which both were present in the nucleus and cytoplasm and was further confirmed by PCR (Figure 1A‐b), or in situ hybridization and FISH (Figure 1A‐c). BC had no protein‐coding potential according to the online Coding Potential Assessment Tool (CPAT), similar to LOC100132707 and lncRNA‐AL589182.3 (Figure S1A). To further confirm BC expression in lung cancer cells, we examined BC expression in a variety of lung cancer cell lines (95C, 95D, PC9, PG‐LH7, PG‐BE1, A549 and H460) and found that BC was highly expressed in all of the cancer cell lines in comparison with BEAS‐2B cells, a non‐tumour bronchial epithelial cell line (Figure 1A‐d). In another pair of lung cancer cell lines with identical genetic backgrounds, BC expression was higher in highly metastatic PG‐BE1 cells than in less metastatic PG‐LH7 cells. Those results suggested that BC expression was associated with the metastatic potential of lung cancer cells.

### 3.2 BC promotes lung cancer proliferation and metastasis

To investigate the role of BC in LUAD progression, we grouped the LUAD dataset from TCGA by their high and low BC expression levels and performed GSEA on the gene expression signatures of these samples, and found that the gene expression signatures of BC high expression group were significantly enriched with functions related to GO: epidermal growth factor receptor signalling pathway and KEGG:_EGFR‐TKI resistance (Figure S1B). Compared to non‐tumour tissues, the mRNAs in LUAD were also enriched in EGFR signalling pathway. In addition, we also performed canonical gene‐enrichment analysis of all BC‐related mRNAs in the TANRIC database.^28^ Gene ontology analysis indicated that BC‐related genes were enriched with functions related to regulation of cytokine production and immune response (Figure S1C). Next, we overexpressed BC in 95C cells (Figure 1B‐a). The BC overexpression enhanced cell growth (Figure 1B‐b) and colony formation in 95C cells (Figure 1B‐c), reduced apoptosis in A549 cells (Figure 1C) and accelerated cell migration, invasion and wound healing in 95C or 95D cells (Figure S2A–C). To investigate the effects of BC expression in lung cancer cells in vivo, we injected A549 cells stably expressing BC into the tail veins of nude mice. Four weeks after injection, the mortality rate among the mice injected with BC‐overexpressing cells (6/8) was higher than that of mice injected with control cells that were transfected with mock (2/8). The numbers of metastasis foci in the lungs and liver were also significantly higher in the mice injected with BC‐expressing cells than in the control group (Figure 1D), suggesting that BC promoted LUAD progression by enhancing tumour cell proliferation and metastasis.

### 3.3 Lung cancer cells overexpressing BC are resistant to EGFR‐TKIs

**FIGURE 2:**
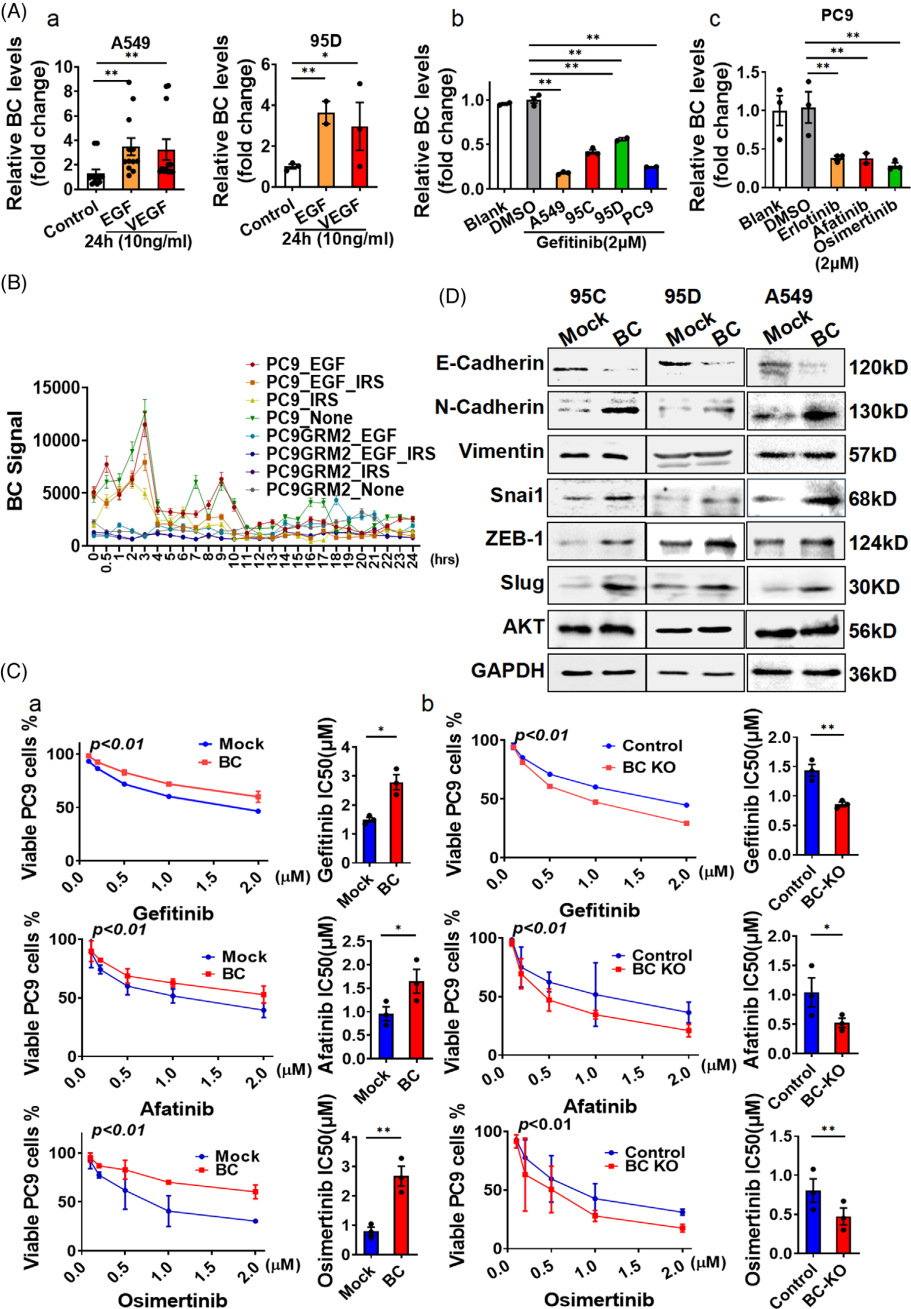
BC enhanced resistance to EGFR‐tyrosine kinase inhibitors (EGFR‐TKIs) by activating the epithelial–mesenchymal transition. (A) (a) BC levels were determined by RT‐qPCR in A549 and 95D cells treated with EGF or VEGF. (b) BC levels were determined by RT‐qPCR in A549, 95C, 95D and PC9 cells treated with 2 μm/L gefitinib (24 h), vehicle or blank control. (c) BC expression was measured in PC9 cells treated with 2 μm/L erlotinib, afatinib or osimertinib. (B) BC expression was measured in gefitinib‐sensitive PC9 cells and gefitinib‐resistant PC9G cells from public microarray dataset GSE34228. IRS, gefitinib‐treatment. PC9GRM2, gefitinib‐resistance cell line. (C) (a and b) Viability of (a) BC‐overexpressing and (b) BC knockout PC9 cells was measured by the MTT assay after treatment with various concentrations of gefitinib (upper), afatinib (middle) or osimertinib (bottom) for 24 h. The IC50 of the EGFR‐TKIs was also measured. (D) The expression of cadherin, vimentin, Snai1, Slug, ZEB‐1 and AKT were determined by Western blot in 95C, 95D and A549 cells with BC overexpression. Data are represented as mean ± SEM of at least three independent experiments. *p < .05, **p < .01

We then treated A549 and 95D cells with EGF or VEGF (10 ng/ml) for 24 h and found that the expression of BC was strongly enhanced following treatment (Figure 2A‐a). To further investigate the effect of EGFR on BC expression, we treated A549, 95C, 95D and PC9 cells with the EGFR‐selective inhibitor gefitinib and found that BC expression levels in all the cell lines were significantly reduced after the treatment (Figure 2A‐b). Treatment of the same cell lines with erlotinib, afatinib or osimertinib similarly reduced the expression levels of BC (Figure 2A‐c). Analysis of BC expression in gefitinib‐resistant PC9G cells and gefitinib‐sensitive PC9 cells, in the public microarray dataset GSE34228,^29^ showed that BC expression was increased in the gefitinib‐resistant cells (PC9GR) compared with that in the gefitinib‐sensitive cells PC9 after EGF treatment (Figure 2B). But the overexpression of BC did not cause EGFR phosphorylation (Figure S2D). In addition, BC‐overexpressing PC9 cells showed low sensitivity to gefitinib, afatinib and osimertinib; increased viability; and increased IC50 values compared with the control cells (Figure 2C‐a). By contrast, PC9 cells with BC knockout showed decreased viability after exposure to osimertinib, gefitinib or afatinib compared with the control cells (Figure 2C‐b).

To understand the bioactivity of BC, we mapped the BC molecule into four domains according to the predicted secondary structure and constructed truncated mutants (D1–D4) (Figure S3A). The D3 and D4 mutants promoted cell growth similarly to the full‐length BC, but the D1 and D2 mutants had almost no growth‐promoting effects (Figure S3B), suggesting that the 330–719 fragment in domain 3 was important for the growth‐promoting activity. Therefore, we compared the scratches and migration capabilities of 95D cells expressing BC domain 2 or domain 3 and confirmed the effect of 330–719 fragment in domain 3 (Figure S3C,D). To further clarify the role of BC in EGFR‐TKI resistance, we performed correlation analysis using data from the TCGA database. The results showed that N‐cadherin expression was closely correlated with BC expression, whereas the expressions of vimentin, Snai1, ZEB1 and E‐cadherin were weakly correlated with BC expression (Figure S4A). We then investigated the impact of BC on EMT in lung cancer cells and found that the expression levels of Slug, N‐cadherin, Snai1 and ZEB1 were significantly elevated in BC‐overexpressing 95C, 95D, A549 or PC9 cells, whereas that of E‐cadherin was robustly reduced (Figures 2D and S4B). We found that the BC‐related mRNAs were significantly enriched in HALLMARK_Epithelial_Mesenchymal_Transition pathway (Figure S4C). In addition, in the GSEA of BC‐overexpressed 95C and 95D cells RNA‐Seq data, the BC‐induced differential expressions were also significantly enriched in EMT pathway (Figure S4D). These connected BC to the EMT process in LUAD.

### 3.4 BC regulates EMT in collaboration with inositol monophosphatase domain containing 1 (IMPAD1)

**FIGURE 3:**
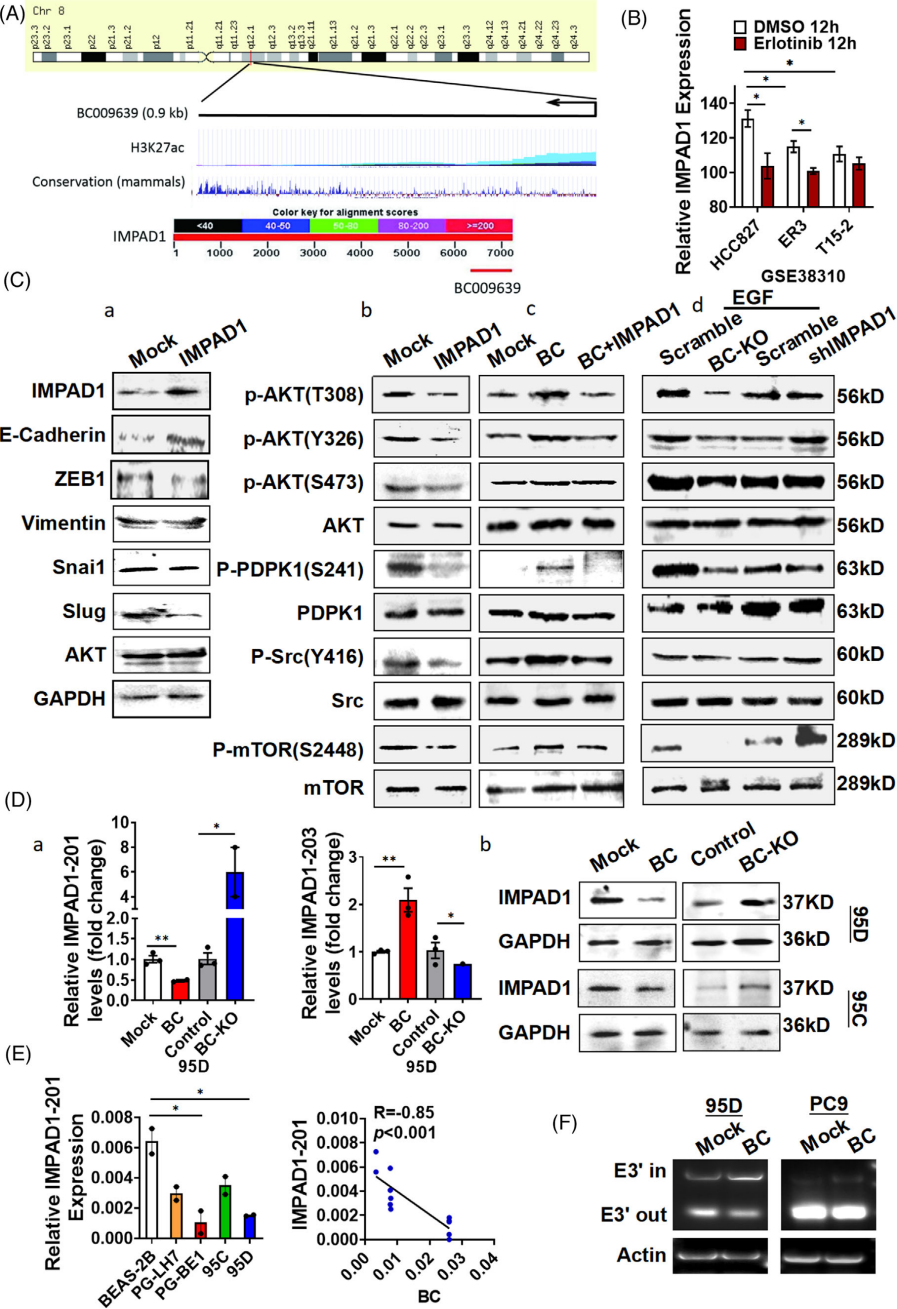
BC enhanced the epithelial–mesenchymal transition by regulating inositol monophosphatase domain containing 1 (IMPAD1) alternative splicing. (A) Schematic representation of BC and UCSC Genome Browser tracks depicting H3K27ac chromatin immunoprecipitation (ChIP)‐seq coverage (upper) and mammalian conservation in human cell lines (middle). Schematic diagram of the complementation between the *IMAPD1* nucleotide sequence and BC determined by BLAST (lower). (B) IMPAD1 expression analysis of cells resistant or sensitive to erlotinib was performed using public microarray dataset GSE38310. (C) (a) IMPAD1, cadherin, vimentin, Snai1, ZEB‐1, Slug and AKT were detected by Western analysis in 95D and A549 cells overexpressing IMPAD1; (b) phosphorylation of AKT, PDPK1, Src and mTOR in 95D cells overexpressing IMPAD1 was measured; (c and d) phosphorylation levels of AKT, PDPK1, Src and mTOR were determined by Western blot in (c) 95D cells with BC and IMPAD1 co‐transfection and (d) 95D cells with BC knockout or IMPAD1 knock‐down after 10 ng/ml EGF treatment. (D) (a) The IMPAD1‐201 and IMPAD1‐203 splice variants were detected by RT‐qPCR in 95D cells after BC overexpression or knock‐down; (b) IMPAD1 expression was detected by Western analysis in 95D and 95C cells with BC overexpression or knockout. (E) IMPAD1‐201 expression was measured by RT‐qPCR in BEAS‐2B, PG‐LH7, PG‐BE1, 95C and 95D cells (left). Pearson correlation between BC and IMPAD1‐201 expression levels is shown (right). (F) The splicing of IMPAD1‐203 exon 3′ in 95D and PC9 cells was detected by PCR. Data are represented as mean ± SEM of at least three independent experiments. *p < .05, **p < .01

The gene encoding BC is actively transcribed from chromosome 8, as indicated by histone lysine acetylation analysis (Figure 3A upper). The nucleotide sequence of BC is partially complementary to the 3′ untranslated region (UTR) of the IMPAD1 (also known as BPNT2 [3′(2′), 5′‐bisphosphate nucleotidase 2]) mRNA (Figure 3A lower). Interestingly, when treated with 1 μm erlotinib for 12 h, the IMPAD1 mRNA levels were significantly lower in erlotinib‐resistant ER3 and T15‐2 cells than in sensitive cells HCC827 (Figure 3B) in the public dataset GSE38310. IMPAD1 overexpression, however, inhibited wound healing in 95D cells but had no effect on cell migration, which was different from the observed BC effects (Figure S5A). In addition, IMPAD1‐overexpressing PC9 cells showed high sensitivity to gefitinib, afatinib or osimertinib compared with the control cells (Figure S5B), which was similar to the PC9 cells with BC knockout. We further investigated the influence of IMPAD1 on the EMT process and noted that E‐cadherin expression in 95D and A549 cells overexpressing IMPAD1 was elevated compared with that in the control cells, whereas Slug expression was reduced (Figures 3C‐a and S5C), suggesting that IMPAD1 played an important role in MET process. Furthermore, the phosphorylation of AKT T308, Y326 and S473; PDPK1 S241; Src Y416; and mTOR S2448 was reduced in the IMPAD1‐overexpressing cells (Figures 3C‐b and S5D). By contrast, in cells with BC overexpression, the phosphorylation of AKT T308, Y326 and S473; PDPK1 S241; Src Y416; and mTOR S2448 was greatly enhanced compared with that in the control cells (Figure 3C‐c), indicating that BC stimulated EMT process. When cells were co‐transfected with BC and IMPAD1, the effects of BC on AKT, PDPK1, Src and mTOR phosphorylation were attenuated (Figure 3C‐c), suggesting that the collaboration existed between BC and IMPAD1 in regulation of EMT. Furthermore, EGF‐stimulated phosphorylation of AKT (S473, T308 and Y326), PDPK1 (S241), Src (Y416) and mTOR (S2448) were reduced after BC knockout (Figure 3C‐d), linking BC action to EGFR signalling. However, the D2 mutant, which lacked the 330–719 fragment of BC, enhanced E‐cadherin and IMPAD1 expression, whereas it reduced Slug expression and Src Y416 and PDPK S241 phosphorylation, which was completely different from the effects of intact BC (Figure S5E). This demonstrated that the action of BC was sequence specific in the regulation of EMT, which might involve the IMPAD1 collaboration.

### 3.5 BC regulates the expression of IMPAD1 transcript variants

The results of in situ hybridization indicated that lncRNA BC was mainly distributed in the cytoplasm around the nucleus (Figure 1A‐c), suggesting that it might be associated with the regulation of RNA processes. We then measured IMPAD1 transcript variants in BC‐overexpressing cells and found that BC robustly increased IMPAD1‐203 expression and reduced IMPAD1‐201 expression (Figure 3D‐a). The IMPAD1‐201 variant has five exons and encodes the IMPAD1 protein. The IMPAD1‐203 variant does not encode any protein, because it lacks exon 1, part of exon 2 and exon 5, but it contains extra nucleotides in exon 3′ spliced from intron 3 (Figure S6A upper). Correspondingly, BC overexpression drastically reduced IMPAD1 protein levels (Figure 3D‐b). Furthermore, IMPAD1‐201 levels were significantly lower in PG‐LH7, PG‐BE1, 95C and 95D lung cancer cells than in BEAS‐2B cells (Figure 3E left). There was a significant correlation (*R* = −.85) between IMPAD1‐201 and BC expression levels in these lung cancer cells (Figure 3E right, *p* < .001). To investigate the role of BC in IMPAD1 splicing, we performed RT‐PCR using splice junction‐specific primers (203‐E3′‐F and 203‐E3′‐R) (Figure S6A lower) in BC‐overexpressing 95D cells. The results showed that following BC overexpression, the levels of IMPAD1‐203 transcripts including exon 3′ (E3′ in) increased, whereas the levels of the IMPAD1‐203 transcripts lacking exon 3′ (E3′ out) decreased (Figure 3F). We further performed RNA‐Seq analysis on BC‐overexpressing lung cancer cell lines to validate the effect of BC on IMPAD1 splice variants (Figure S6B,C). These suggested that BC was involved in regulation of IMPAD1 alternative splicing (AS).

### 3.6 BC regulates IMPAD1 alternative splicing via hnRNPK

**FIGURE 4:**
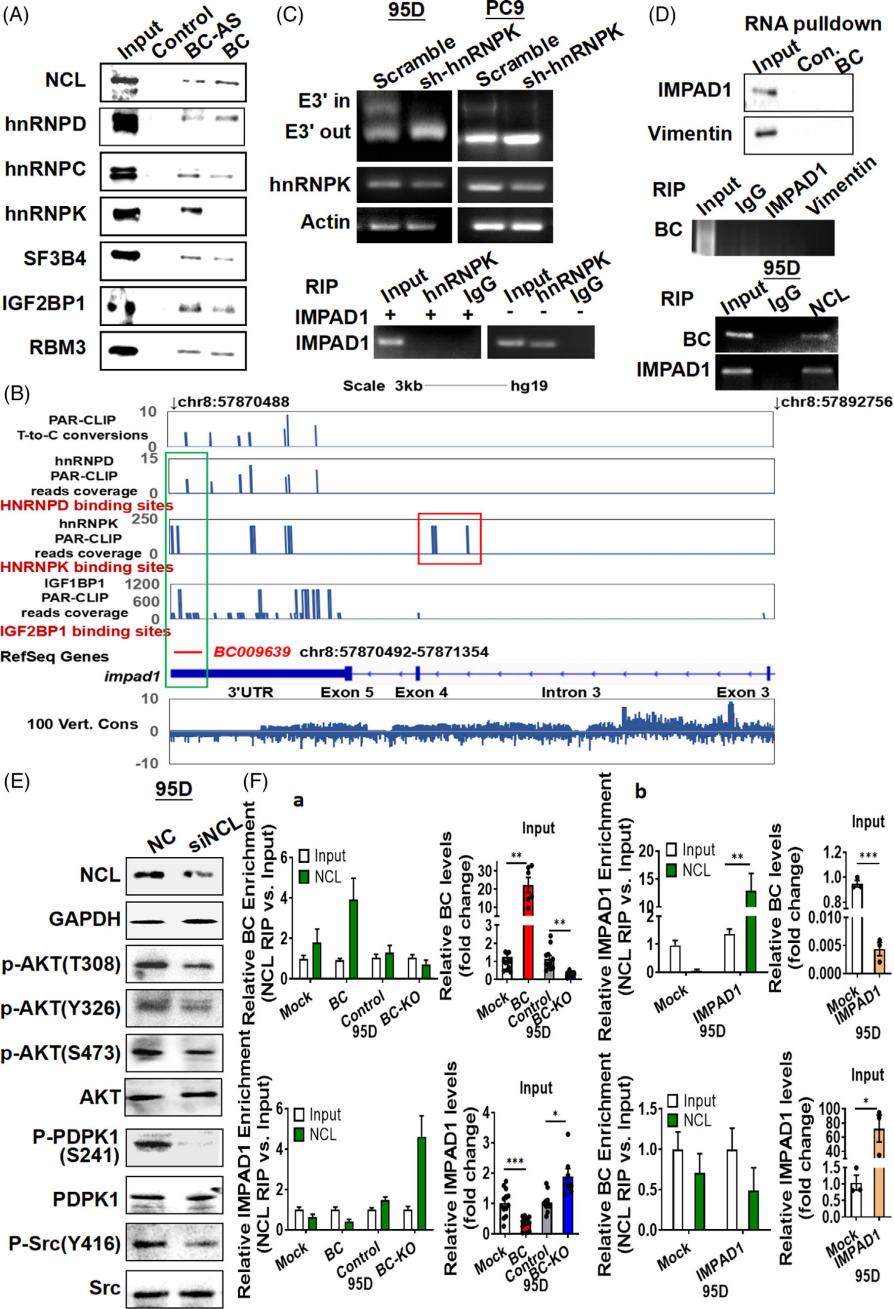
BC regulates inositol monophosphatase domain containing 1 (IMPAD1) alternative splicing via an interaction with hnRNPK and NCL. (A) Binding of BC to NCL, heterogeneous nuclear ribonucleoprotein (hnRNP), IGF2BP1, SF3B4 and RBM3 was analysed by BC RNA pull‐down assay. (B) Genome browser views of hnRNPD, hnRNPK and IGF2BP2 PAR‐CLIP binding sites and sequence conservation across vertebrates in genomic regions spanning IMPAD1 and BC. The aligned PAR‐CLIP reads are highlighted in BC‐aligned regions and IMPAD1 intron 3 with frame. (C) Splicing of IMPAD1‐203 exon 3′ was detected by PCR in 95D and PC9 cells following hnRNPK knock‐down (upper). RNA immunoprecipitation using the hnRNPK antibody was performed in 95D cells with IMPAD1 overexpression (lower). IgG‐bound RNA was used as a negative control. (D) IMPAD1 or vimentin binding to BC was analysed by BC RNA pull‐down and RNA immunoprecipitation (top, middle). Binding of NCL to BC and IMPAD1 mRNA were detected by RNA immunoprecipitation (bottom). (E) NCL, PDPK1, Src, AKT and their phosphorylation were detected by Western analysis in 95D cells with NCL knock‐down. (F) RNA immunoprecipitation and RT‐qPCR analysis were performed to measure BC and IMPAD1 mRNA binding to NCL protein in 95D cells with BC overexpression or knock‐down (a) or in 95D cells with IMPAD1 overexpression (b). Data are represented as mean ± SEM of at least three independent experiments. *p < .05, **p < .01

To understand the possible mechanism of BC‐mediated regulation, we measured the ability of BC to form a complex with various proteins on a protein microarray. A total of 49 proteins bound to BC (Table S3). Gene ontology analysis showed that most of the BC‐binding proteins were associated with RNA splicing and processing (Figure S7A). In a separate RNA pull‐down and mass spectrometry analysis, a total of 138 proteins were found to interact with BC (Figure S7B). Seven proteins were common to both the protein microarray and the mass spectrometry results: hnRNPC, hnRNPD, hnRNPK, hnRNPUL1, PABPC1, nucleolin (NCL) and peroxiredoxin‐1 (RDX). We further conducted additional RNA pull‐down assays to confirm the binding of those proteins to BC and IMPAD1 transcripts. The results showed that the BC antisense complex had a stronger affinity for hnRNPK and hnRNPC than the BC complex (Figure 4A). By contrast, the BC complex had a stronger affinity than the BC antisense complex for hnRNPD and NCL. Both BC and BC antisense complexes also interacted with SF3B4, IGF2BP1 and RBM3 but did not pull down MGMT, hnRNPCL1, hnRNPUL, Argonaute 2 and U1 small nuclear ribonucleoprotein (snRNP) (Figure S7C).

We further investigated the binding sites of the protein complexes by RNA crosslinking immunoprecipitation followed by RT‐PCR (CLIP‐PCR) in HEK‐293 cells. The analysis showed that hnRNPK interacted specifically with intron 3 and 3′UTR of the IMPAD1‐201 pre‐mRNA, hnRNPD and IGF1BP1 interacted with BC and the 3′UTR of IMPAD1‐201, whereas hnRNPC, SF3B4 and RBM3 did not (Figure 4B). Therefore, we knocked down hnRNPK in 95D or PC9 cells and detected IMPAD1 transcript variants using the 203‐E3′‐F/R splice junction‐specific primers. We could detect the ‘E3′ out’ transcript variant but not the ‘E3′ in’ variant in the hnRNPK knock‐down cells (Figure 4C upper), suggesting that hnRNPK was required for IMPAD1‐203 AS. Next, we transfected 95D cells with an exogenous IMPAD1 expression plasmid, that did not contain an intron sequence, and used RIP experiments to verify the binding of IMPAD1 to hnRNPK. As expected, the exogenous IMPAD1 lacking the intron sequence of IMPAD1‐201 was unable to bind to hnRNPK (Figure 4C lower). To determine whether the expression of hnRNPK was associated with LUAD resistance to EGFR‐TKIs, we further analysed public datasets from TCGA and GEO and found that the expression levels of hnRNPK were significantly higher in gefitinib‐resistant lung cancer cell lines compared with their parental cells and strongly associated with poor overall survival (Figure S7D), indicating that hnRNPK was involved in LUAD resistance to EGFR‐TKIs.

### 3.7 BC forms a splicing complex with IMPAD1 by interacting with NCL

Next, we conducted additional RNA pull‐down assays to determine proteins that involved in the forming of BC‐mediated splicing complex for IMPAD1 as BC did not strongly bind to hnRNPK. Neither vimentin nor IMPAD1 could be identified in the complexes pulled down by BC (Figure 4D top and middle). We noted, however, that NCL bound to BC more robustly than to the BC antisense RNA (Figure 4A). Phospho‐NCL (T76 or T84) was not found in the complex pulled down by BC (Figure S7C upper). RIP further confirmed that both BC and the IMPAD1 transcripts were present in the complex immunoprecipitated by the NCL antibody (Figure 4D bottom). We therefore knocked down NCL by RNA interference and observed that the phosphorylation of Src Y416, PDPK S241 and AKT was attenuated as a result of the NCL knock‐down (Figure 4E), which was similar to the effect of IMPAD1 overexpression (Figure 3C‐b). This indicated that NCL played an important role in IMPAD1 splicing complex (Figure S7C lower). To further investigate the influence of BC on NCL binding of IMPAD1 mRNA, we overexpressed and knocked down BC in lung cancer cells and detected the levels of BC RNA in the NCL complex (Figure 4F‐a). Overexpression of BC enhanced BC–NCL binding but attenuated IMPAD1 mRNA–NCL binding. Conversely, BC knockout or IMPAD1 overexpression attenuated BC–NCL binding but enhanced IMPAD1 mRNA–NCL binding (Figure 4F‐b). The NCL protein level did not change after IMPAD1 or BC overexpression (data not shown). This suggested that BC required NCL interaction to form the splicing complex with IMPAD1 transcript.

### 3.8 BC expression is associated with poor LUAD clinical outcomes

**FIGURE 5:**
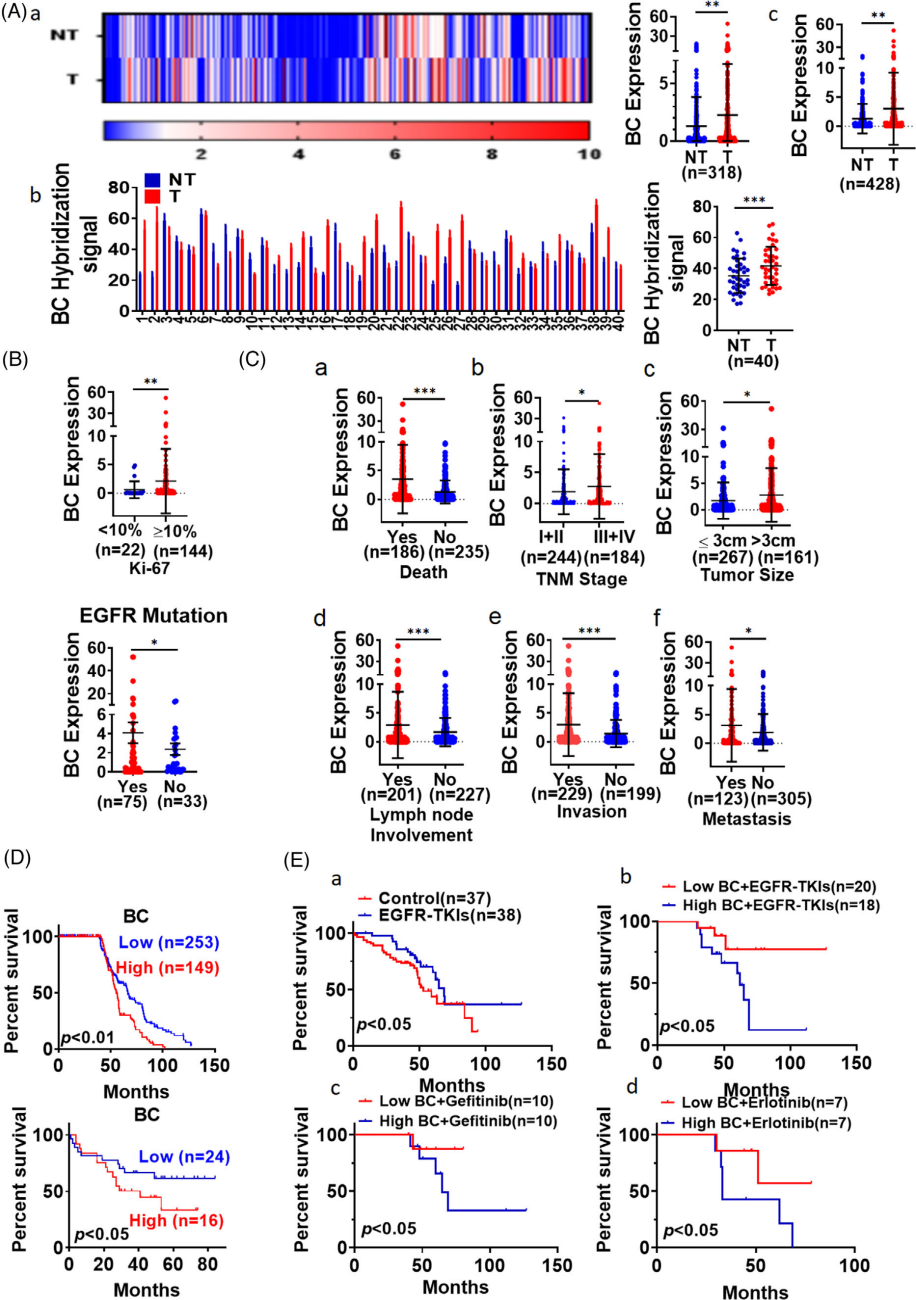
BC was highly expressed in lung adenocarcinoma (LUAD) tissues. (A) (a) Differential BC expression between LUAD (T) tissues and adjacent non‐tumour (NT) tissues from 318 patients was determined by RT‐qPCR; (b) BC expression was measured by in situ hybridization in LUAD tissues and adjacent non‐tumour tissues from 40 patients; (c) BC expression analysis in the combined cohort (*n* = 428). (B) BC levels in LUAD tissues were compared between patients with different positive rate of Ki‐67 staining (upper). BC levels were measured in lung cancer tissues with and without EGFR mutation from 428 patients with LUAD (lower). (C) BC levels in LUAD tissues were compared between patients with different (a) survival, (b) TNM stage, (c) tumour size, (d) lymph node involvement, (e) invasion and (f) remote metastasis. Data are representative of at least three independent experiments. *p < .05, **p < .01. (D) Overall survival among 402 patients with LUAD was analysed according to BC expression levels measured by RT‐qPCR (upper). Kaplan–Meier analysis of 40 patients with LUAD showed differential survival corresponding to BC in situ hybridization signal levels (lower). (E) Kaplan–Meier analysis of overall survival of patients with LUAD that received EGFR‐tyrosine kinase inhibitor (EGFR‐TKI) treatment and conventional chemotherapy (a); Kaplan–Meier analysis of overall survival of patients with LUAD that received EGFR‐TKI treatment based on BC expression (b); overall survival analysis of patients who received treatment with gefitinib (c) or erlotinib (d) based on BC expression

To verify the role of BC in patients, we next analysed BC expression by RT‐qPCR in pairs of tumour and adjacent non‐tumour tissues from a cohort of 318 patients with LUAD. The results showed that BC expression was significantly higher in the tumour tissues than in the adjacent non‐tumour tissues (Figure 5A‐a). We also measured BC expression in a cohort of 40 patients with LUAD by in situ hybridization. The average intensity of hybridization signals in another cohort of 40 patients was significantly stronger in tumour tissues than in non‐tumour tissues (Figure 5A‐b). Data from the TCGA LUAD database also showed that LUAD tissues had elevated BC expression compared with non‐tumour tissues (Figure S8A, *p* < .05). We then measured the expression level of BC in unpaired tumour tissues and non‐tumour tissues from a different cohort of 110 patients with LUAD by RT‐qPCR. In a combined analysis of that cohort and the previous cohort, with a total sample size of 428 (318 + 110), we found that BC expression in the tumour tissues was significantly higher than in the non‐tumour tissues (Figure 5A‐c). BC expression was significantly higher in LUAD tissues with high positive rate of Ki67 staining (>10%) compared to those with low positive rate of Ki67 staining (<10%) (Figure 5B upper), suggesting that BC expression was related to tumour growth. BC expression in the EGFR‐mutated tumours was also significantly higher than that in the non‐EGFR‐mutated LUAD tumours (Figure 5B lower), suggesting BC level association with the EGFR‐mutated proliferation signals. BC expression was also higher among the patients that had died than in those that were still alive at the time of our analysis (Figure 5C‐a, *p* < .001). Patients with TNM stage III or IV disease had higher BC expression than those with stage I or II disease (Figure 5C‐b, *p* < .05). Patients with large tumours (>3 cm) had higher BC expression than those with small (≤3 cm) tumours (Figure 5C‐c, *p* < .05). Patients with lymph node involvement, invasion and metastasis also showed greater BC expression than those without lymph node involvement, invasion and metastasis (Figure 5C‐d–f; *p* < .001). Those results indicated that BC overexpression was closely associated with clinical progress in patients with LUAD. We retrospectively analysed the clinical outcome of these 428 patients who were followed up 10 years for survival analysis although 26 lost during the follow‐up. Kaplan–Meier analysis of 402 patients with LUAD showed that patients with high BC expression had a shorter survival than the patients with low BC expression (Figure 5D upper, ROC cut‐off value = 1.9083, *p* < .001). Among another cohort of 40 patients in whom the BC levels were measured by in situ hybridization, the Kaplan–Meier survival analysis also showed that higher BC levels were associated with poorer overall survival (Figure 5D lower, *p* < .05). Higher BC levels were strongly associated with a poorer overall survival among the patients with LUAD in the TCGA database as well (Figure S8B, *p* < .05). Apparently, the survival outcome of 38 patients receiving EGFR‐TKI treatment was much better than those who received conventional therapy after surgical resection (Figure 5E‐a). Among these 38 patients, these with low BC expression levels achieved significantly longer survival than those with high BC levels (Figure 5E‐b). Moreover, under the same EGFR‐TKI treatment regimen, the survival outcome of patients with high BC expression was still worse than that of patients with low BC expression (Figure 5E‐c,d). These indicated that high BC expression reduced the efficacy of EGFR‐TKI therapy. Similarly, compared with normal tissues, BC were robustly elevated in kidney renal clear cell carcinoma and stomach adenocarcinoma (Figure S8C).

## 4 DISCUSSION

LncRNAs are important in key biological processes, including gene expression and protein function regulation. We investigated the lncRNA profiles of lung cancer cell lines and identified that BC was highly expressed in cell lines with high metastatic potential. Analysis of tumours and adjacent normal tissues from 428 patients with lung cancer revealed that the expression of BC was significantly higher in the tumours than in the adjacent normal tissues. BC expression was negatively correlated with patient survival and was closely associated with tumour growth and metastasis. In particular, BC overexpressed EGFR mutant patients had poor prognosis for EGFR‐TKI treatment. Indeed, BC promoted not only the wound closure and migration of lung cancer cells, but also lung cancer cell proliferation in vivo. In nude mice, BC promoted the metastasis of ectopically implanted lung cancer cells. Thus, our findings may provide a potential new target for lung cancer diagnosis and therapy.

The molecular mechanism of EGFR‐TKIs resistance has not been well understood,^30^ although it is the major problem for LUAD treatment. In this study, we demonstrated that BC overexpression in lung cancer cells reduced cellular sensitivity to the EGFR‐TKIs gefitinib, afatinib and osimertinib. As well, endogenous BC expression was elevated in gefitinib‐resistant PC9G cells. We further observed that in the cells with BC overexpression, the EMT was activated, and the phosphorylation of AKT Y326, S473 and T308 was significantly enhanced. AKT is generally activated by phosphoinositide binding and loop phosphorylation at T308 by PDPK1, whereas mTOR and c‐Src phosphorylate serine 473 at the carboxy terminus and tyrosine 326 of AKT, respectively. Overexpression of BC appeared to activate that pathway.

Bioinformatics analysis showed that the sequence of BC is complementary to the 3′UTR of IMPAD1 mRNA in natural antisense. IMPAD1, an inositol phosphatase protein, can catalyse inositol–phosphate hydrolysis to inositol and phosphoadenosine–phosphate hydrolysis to adenosine monophosphate.^31^ IMPAD1 gene mutation leads to joint cartilage dysplasia^32^ and Catel–Manzke syndrome.^33^ Aberrant overexpression of IMPAD1 was observed in breast cancer with Myc oncogene co‐amplification,^34^ but the precise role of IMPAD1 in cancer is still unknown. We observed that IMPAD1 suppressed EMT by promoting E‐cadherin expression and inactivating AKT. Elevated IMPAD1 levels resulted in increased hydrolysis of inositol phosphates that are the activators of PDPK1. Hydrolysis of inositol phosphates led to PDPK1 suppression which prevented the activation of AKT via phosphorylation at T308.

Overexpression of BC reduced the level of the protein‐coding IMPAD1‐201 transcript variant and increased the level of the non‐coding IMPAD1‐203 transcript variant in lung cancer cells. BC facilitated AS, in which part of intron 3 was spliced into exon 3′ of IMPAD1‐203, whereas exon 1 was skipped. AS is largely regulated by *trans*‐acting protein factors.

Using protein microarrays, mass spectrometry and RNA pull‐down assays, we observed that BC and the 3′UTR of IMPAD1 simultaneously interacted with the RNA binding proteins NCL, hnRNPD, RBM3, SF3B4 and IGF2BP1, whereas hnRNPK mainly bound to IMPAD1. PAR‐CLIP data verified that hnRNPK, hnRNPD and IGF2BP1 bound to the 3′UTR of IMPAD1, but only hnRNPK bound to intron 3 of IMPAD1. AS of pre‐mRNAs to produce structurally and functionally distinct mRNA and protein variants is a key molecular mechanism in the regulation of gene expression and transcriptomic diversity in eukaryotic cells.^35^, ^36^, ^37^, ^38^ More than 95% of the transcripts of human genes with multiple exons undergo AS. The resulting splice variants are variably expressed among different cells and tissues.^39^ Hence, the dysregulation of AS underlies many human diseases.^40^ A recent global analysis of lung cancer RNA‐Seq datasets suggested that aberrant AS patterns were associated with patient survival in LUAD and lung squamous cell carcinoma.^41^ The results of another study indicated that AS of TKI targets, such as Met exon 14 skipping, might modify patient responses to therapy.^42^ Results in this study demonstrated that lncRNA BC mediated IMPAD1 AS leading to EMT activation and induced LUAD to resist EGFR‐TKI.

**FIGURE 6:**
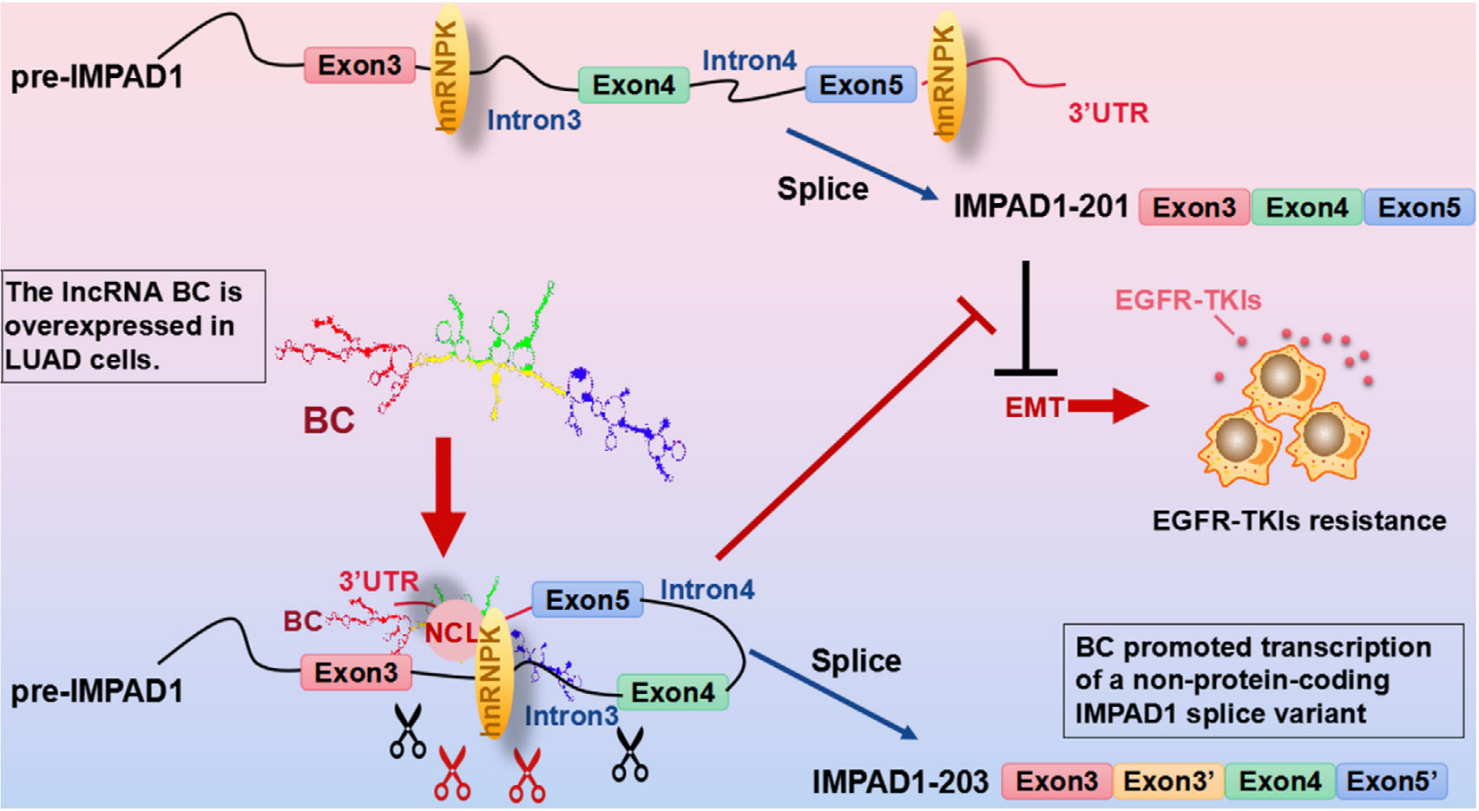
Long non‐coding RNA (LncRNA) BC mediates the resistance to EGFR‐tyrosine kinase inhibitor (EGFR‐TKI) in lung cancer cells via regulating inositol monophosphatase domain containing 1 (IMPAD1) alternative splicing and epithelial–mesenchymal transition (EMT) process.

AS is regulated by *trans*‐acting protein factors, which are primarily RNA binding proteins, such as the snRNPs, heterogeneous nuclear ribonucleoproteins (hnRNPs), the serine/arginine‐rich family of nuclear phosphoproteins (SR proteins) and SR‐related proteins. The mechanisms of AS regulation include the RNA–protein interactions between splicing factors and regulatory sites on pre‐mRNAs. *Cis*‐acting elements of exonic/intronic splicing enhancers and silencers^43^, ^44^ are also recognized by RNA binding proteins or splicing factors to compose a common mechanism to set up and maintain AS patterns.^45^, ^46^ In current study, we observed that BC and the 3′UTR of IMPAD1 interacted with the RNA binding proteins NCL and splicing factors hnRNPD and hnRNPK to form the splicing complex. Human hnRNPK is a modular protein consisting of three conserved KH domains and one KI region between KH2 and KH3. The KH domains are used for RNA/DNA binding, and the KI domain is used for protein interaction^47^ to regulate pre‐mRNA splicing.^48^ Recent evidence indicates that hnRNPK also interacts with many non‐coding RNAs directly and plays a central role in RNA splicing and AS.^49^, ^50^ Furthermore, the expression levels of hnRNPK in gefitinib resistant lung cancer cell lines were also elevated according to the GEO database (GSE75309, GSE34228 and GSE38310). These indicated that hnRNPK played important role in BC‐mediated EGFR‐TKIs resistance. Silencing of hnRNPK caused the splice of IMPAD1 to skip exon 3′, suggesting that hnRNPK was required to produce IMPAD1‐203. BC enhanced the production of IMPAD1‐203, linking it to the modulation of AS, which resulted in EMT activation. Endogenous ncRNAs act as natural antisense transcripts (NATs), which can specifically interact with *cis*‐acting elements in pre‐mRNAs through the *cis*‐NAT or *trans*‐NAT form, impacting AS outcomes.^51^ The lncRNA Saf,^52^ EGOT^53^ or Zeb2^54^ are the *cis*‐NATs which are transcribed from the opposite DNA stand at the same locus and bind to the *cis*‐acting element in the pre‐mRNAto recruit splicing factors. In contrast, the lncRNA BC200 is the *trans*‐NAT which binds the *cis*‐acting element in the Bcl‐x pre‐mRNA and recruits hnRNPA2/B1 to Bcl‐x pre‐mRNA. This interaction produces the Bcl‐xL isoform and suppresses the Bcl‐xS isoform in breast tumour cells.^55^ BC suppressed IMPAD1‐201 transcript variant but promoted the non‐coding IMPAD1‐203 transcript variant through AS. BC not only recruited RNA binding proteins (NCL, IGF2BP1) or splicing factors (hnRNPK) but also regulated the assembly of the splicing‐regulator complex by forming RNA–RNA duplexes to modulate pre‐mRNA processing (Figure 6). HnRNPK binds the C‐rich motifs outside Alu elements, which is the most common short interspersed elements (SINE) in the human. Sequences enriched in Alu are associated with nuclear enrichment in both lncRNAs and mRNAs as SINE‐derived nuclear RNA localization.^56^ BC interaction with hnRNPK might cause its nuclear retention. We also need to consider that BC reduces IMPAD1 protein levels, whereas another possible mechanism by which BC overexpression has the same downstream effect as IMPAD1 siRNA knock‐down is that BC causes RNA decay or translational repression by base pairing with the IMPAD1 mRNA 3′UTR.

## 5 CONCLUSIONS

In summary, our results showed that expression of the lncRNA BC was closely associated with lung cancer cell proliferation and metastasis, poor patient survival and resistance to EGFR‐TKIs. BC bound to NCL and the 3′UTR of IMPAD1 and recruited hnRNPD, hnRNPK and IGF2BP1 to modulate AS of IMPAD1 to produce the non‐protein‐coding IMPAD1‐203 variant and induce EMT process leading to EGFR‐TKI resistance.

## CONFLICTS OF INTEREST

The authors have declared that no conflicts of interest and no competing interest exist.

## Supporting information

Supporting Information

Figure S1 Functional analysis of lncRNA BC
Figure S2 BC promoted lung cancer metastasis
Figure S3 Domain mapping of lncRNA BC
Figure S4 BC enhanced epithelial–mesenchymal transition
Figure S5 IMPAD1 association with EMT in lung cancer cells
Figure S6 BC regulated IMPAD1 alternative splicing
Figure S7 Proteins identified in the BC complex
Figure S8 BC expression analysis in LUAD and other tumours
Table S1 Summary of primers used in the study
Table S2 List of top differential expression LncRNAs (95C vs. 95D, fold change > 3)
Table S3 Potential BC binding proteins
