# Supplementary material for: LncRNA BC promotes lung adenocarcinoma progression by modulating IMPAD1 alternative splicing
Source: Clin Transl Med. 2023 Jan 17;13(1):e1129. doi: 10.1002/ctm2.1129 (PMC9845120; doi:10.1002/ctm2.1129)
Supplement: Supplementary file 1 — Supporting Information [file CTM2-13-e1129-s001.pptx]

## Slide 1
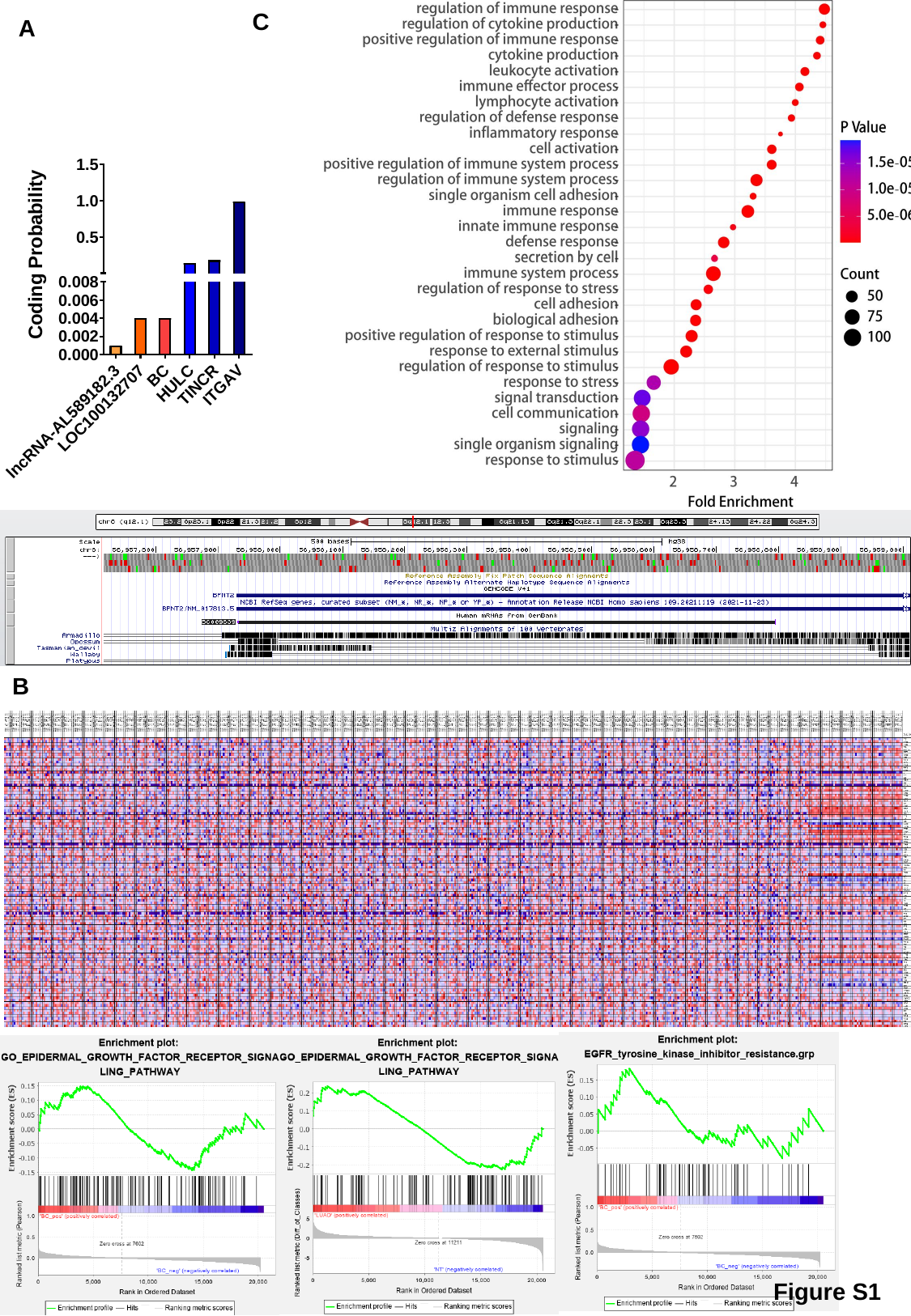

C
A
B
Figure S1

## Slide 2
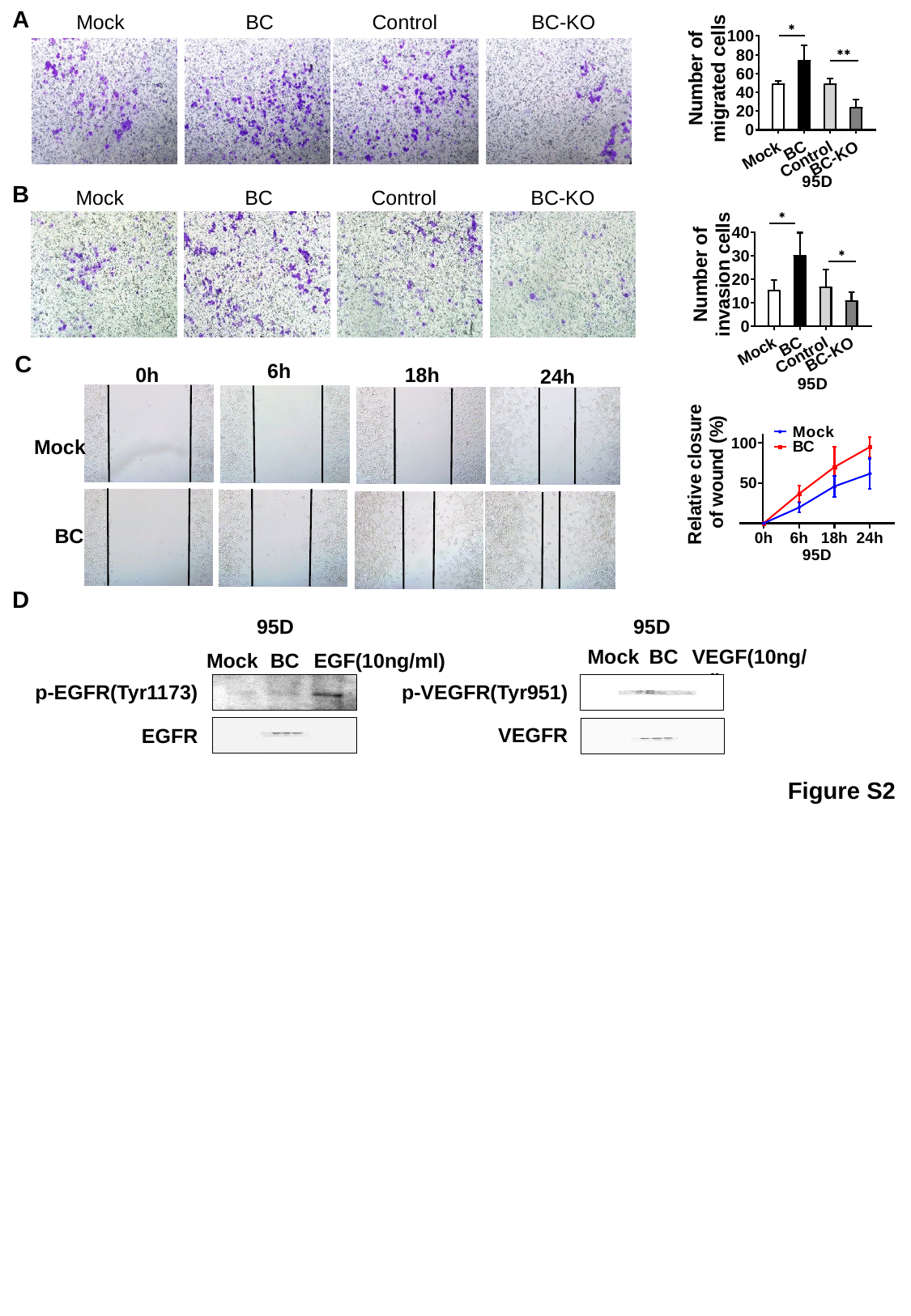

A
Mock
BC
Control
BC-KO
B
Mock
BC
Control
BC-KO
C
6h
18h
0h
24h
Mock
BC
D
95D
95D
Mock
BC
VEGF(10ng/ml)
Mock
BC
EGF(10ng/ml)
p-EGFR(Tyr1173)
p-VEGFR(Tyr951)
VEGFR
EGFR
Figure S2

## Slide 3
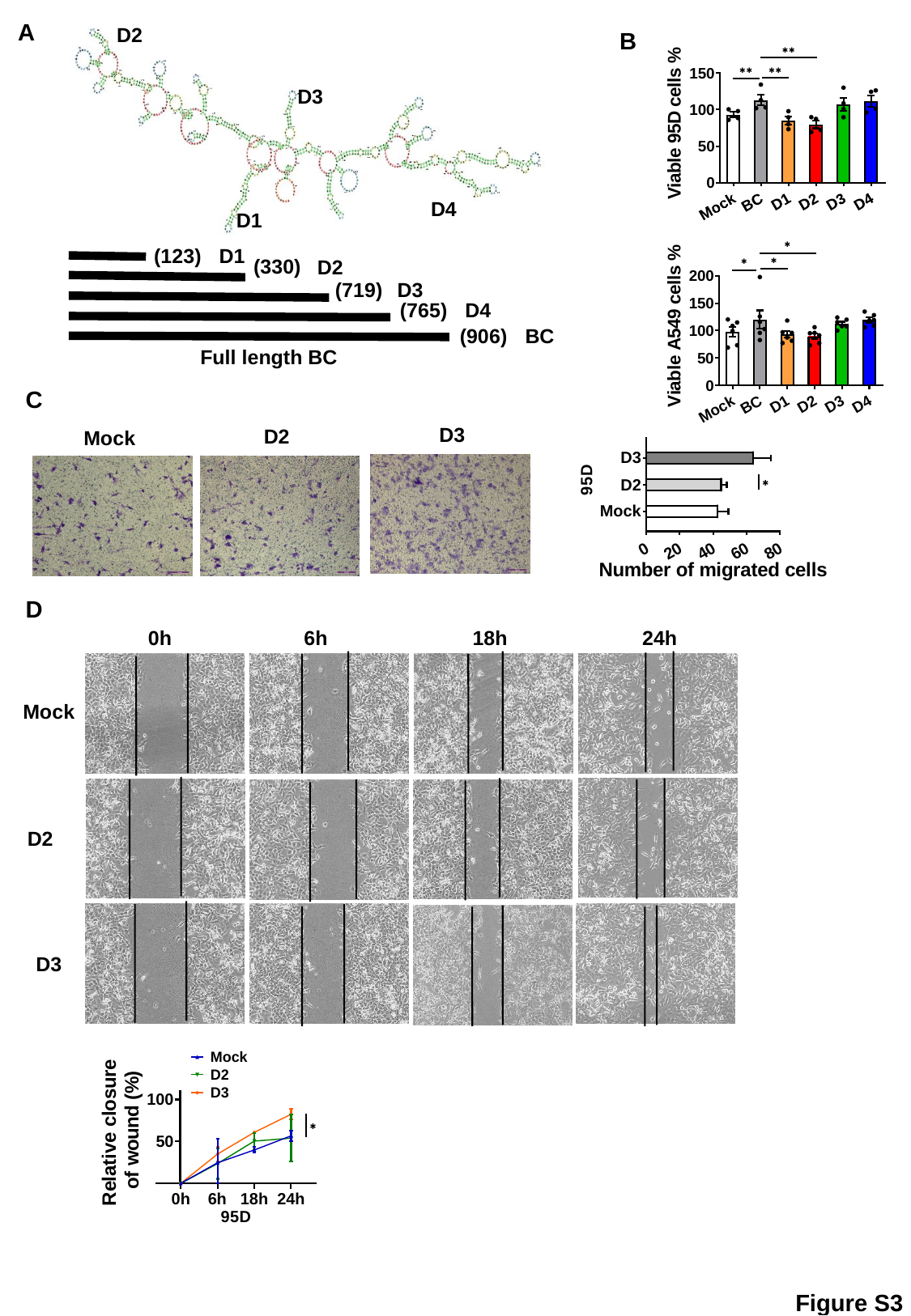

A
D2
D3
D4
D1
B
(123)
D1
(330)
D2
(719)
D3
(765)
D4
(906)
BC
Full length BC
C
D3
D2
Mock
D
0h
6h
18h
24h
Mock
D2
D3
Figure S3

## Slide 4
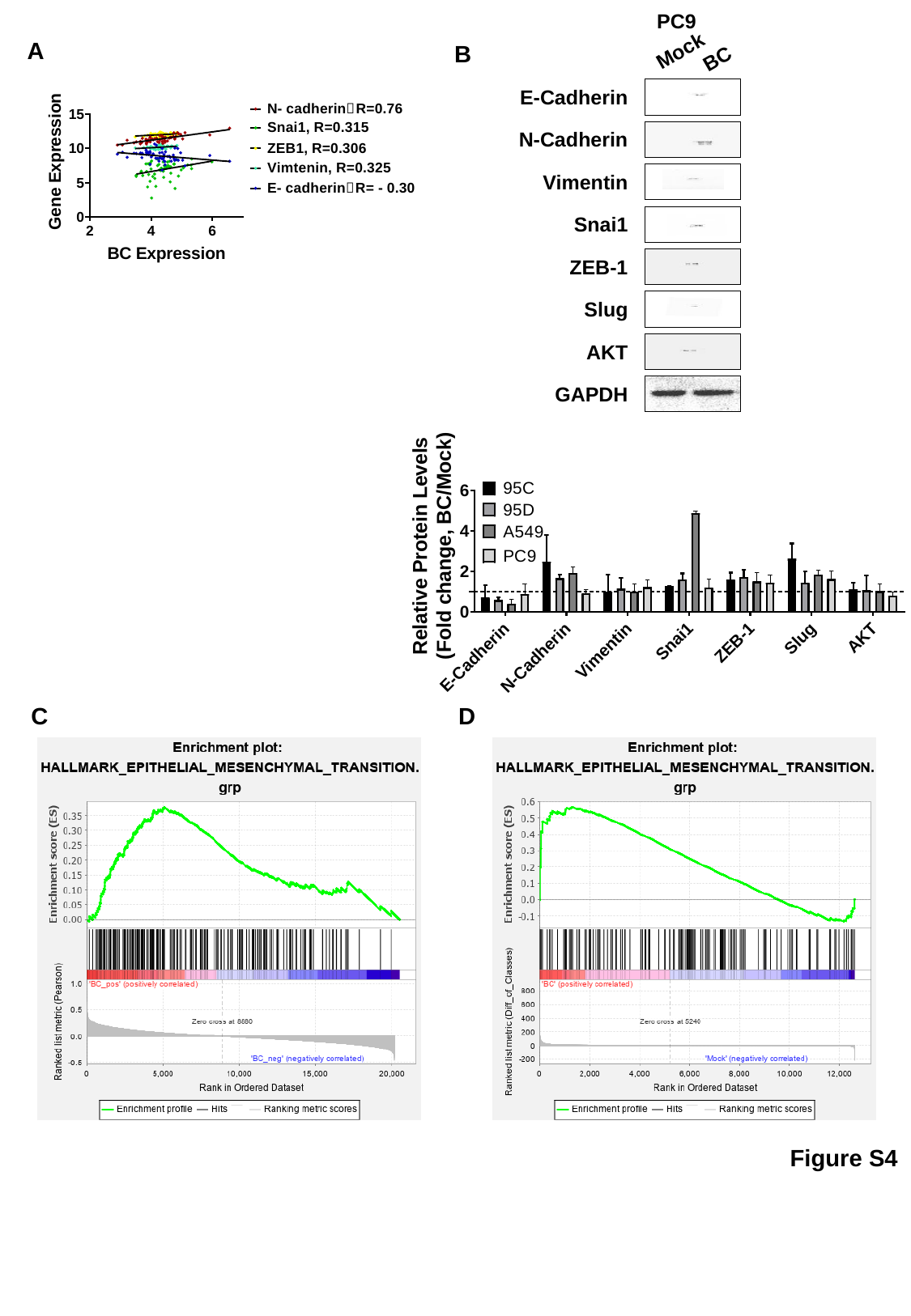

PC9
Mock
BC
E-Cadherin
N-Cadherin
Vimentin
Snai1
ZEB-1
Slug
AKT
GAPDH
A
B
C
D
Figure S4

## Slide 5
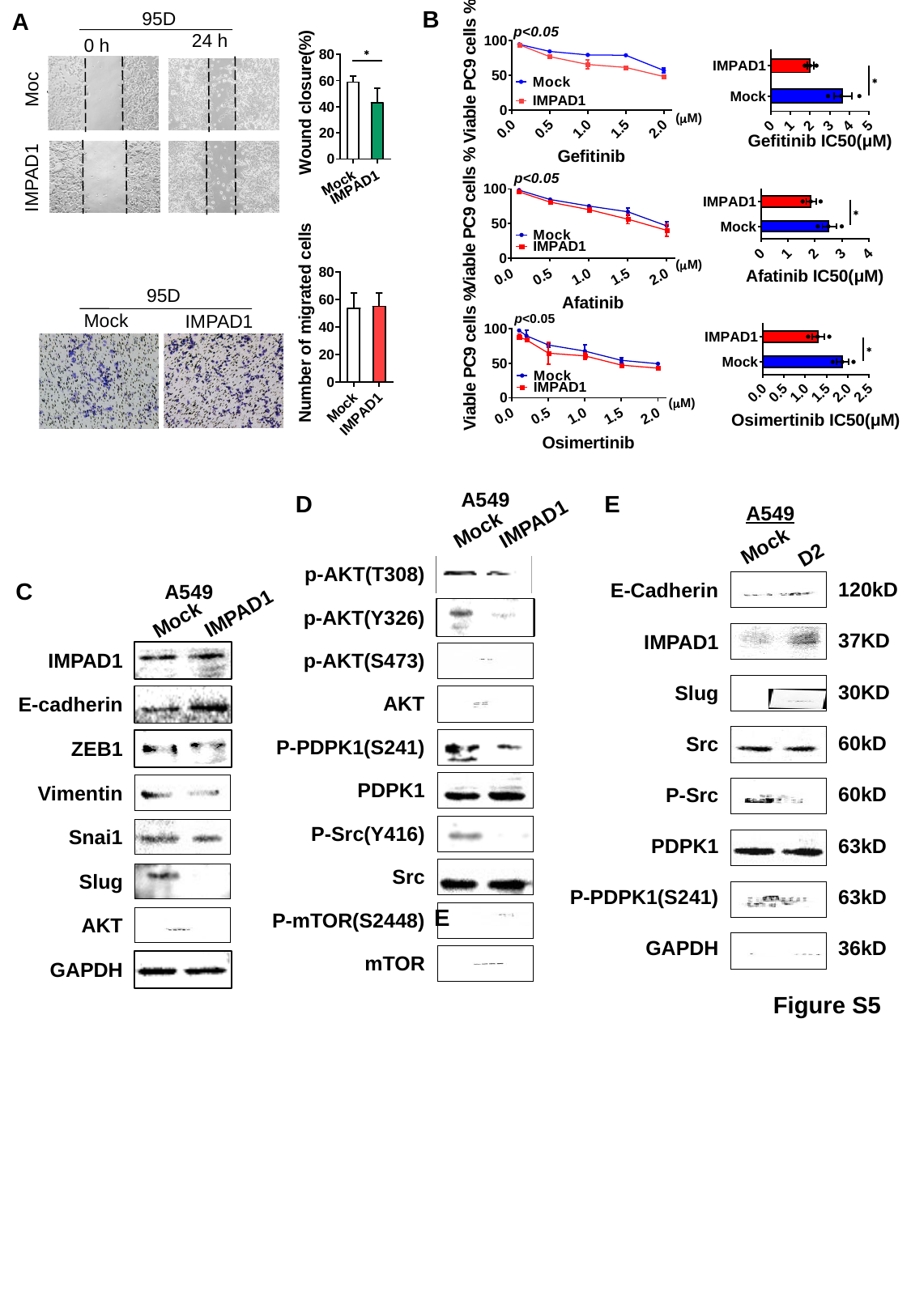

B
A
95D
24 h
0 h
Mock
IMPAD1
95D
Mock
IMPAD1
A549
IMPAD1
Mock
p-AKT(T308)
p-AKT(Y326)
p-AKT(S473)
AKT
P-PDPK1(S241)
PDPK1
P-Src(Y416)
Src
E
P-mTOR(S2448)
mTOR
D
E
A549
Mock
D2
120kD
E-Cadherin
37KD
IMPAD1
30KD
Slug
60kD
Src
60kD
P-Src
63kD
PDPK1
63kD
P-PDPK1(S241)
36kD
GAPDH
C
A549
IMPAD1
Mock
IMPAD1
E-cadherin
ZEB1
Vimentin
Snai1
Slug
AKT
GAPDH
Figure S5

## Slide 6
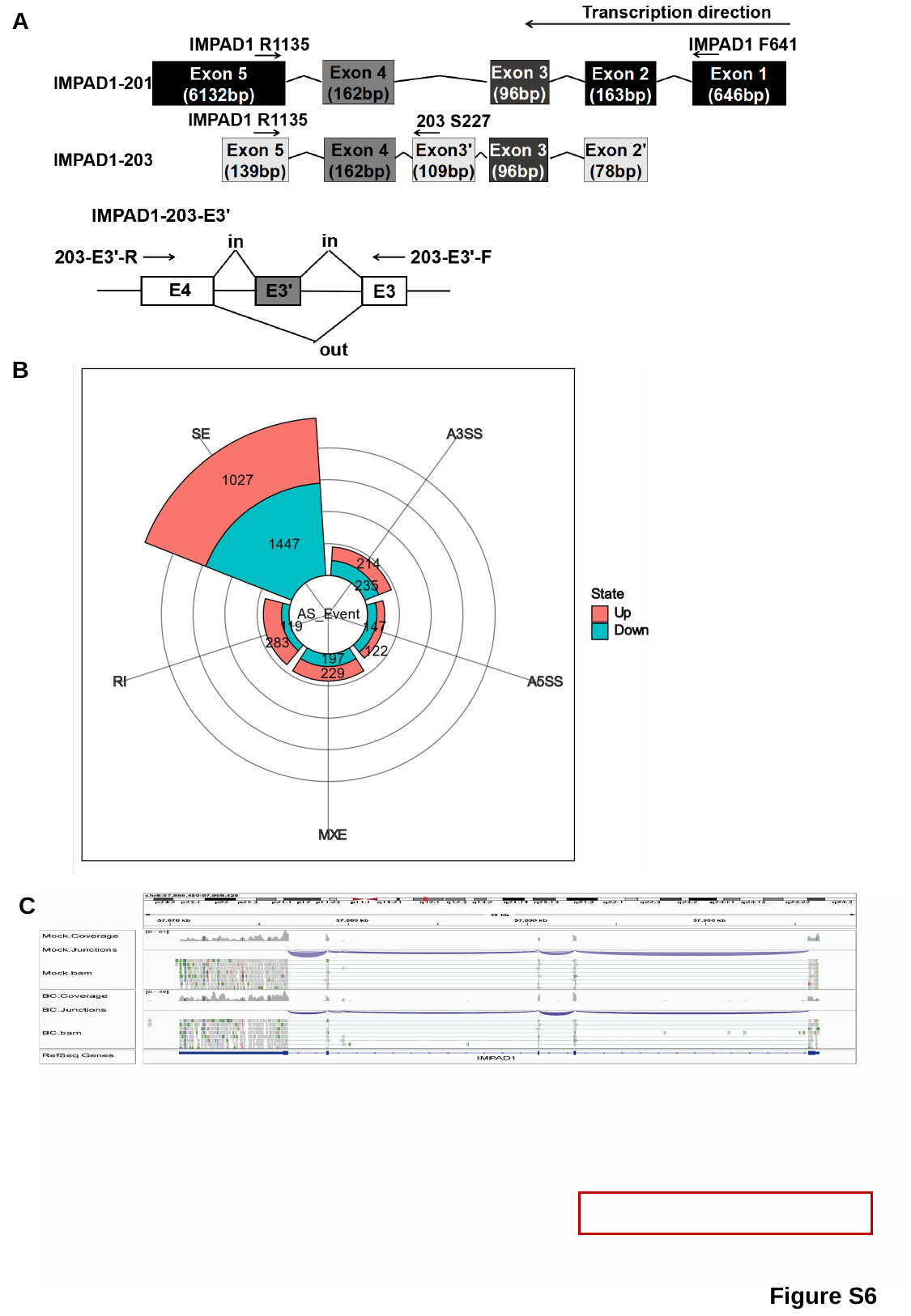

A
B
C
Figure S6

## Slide 7
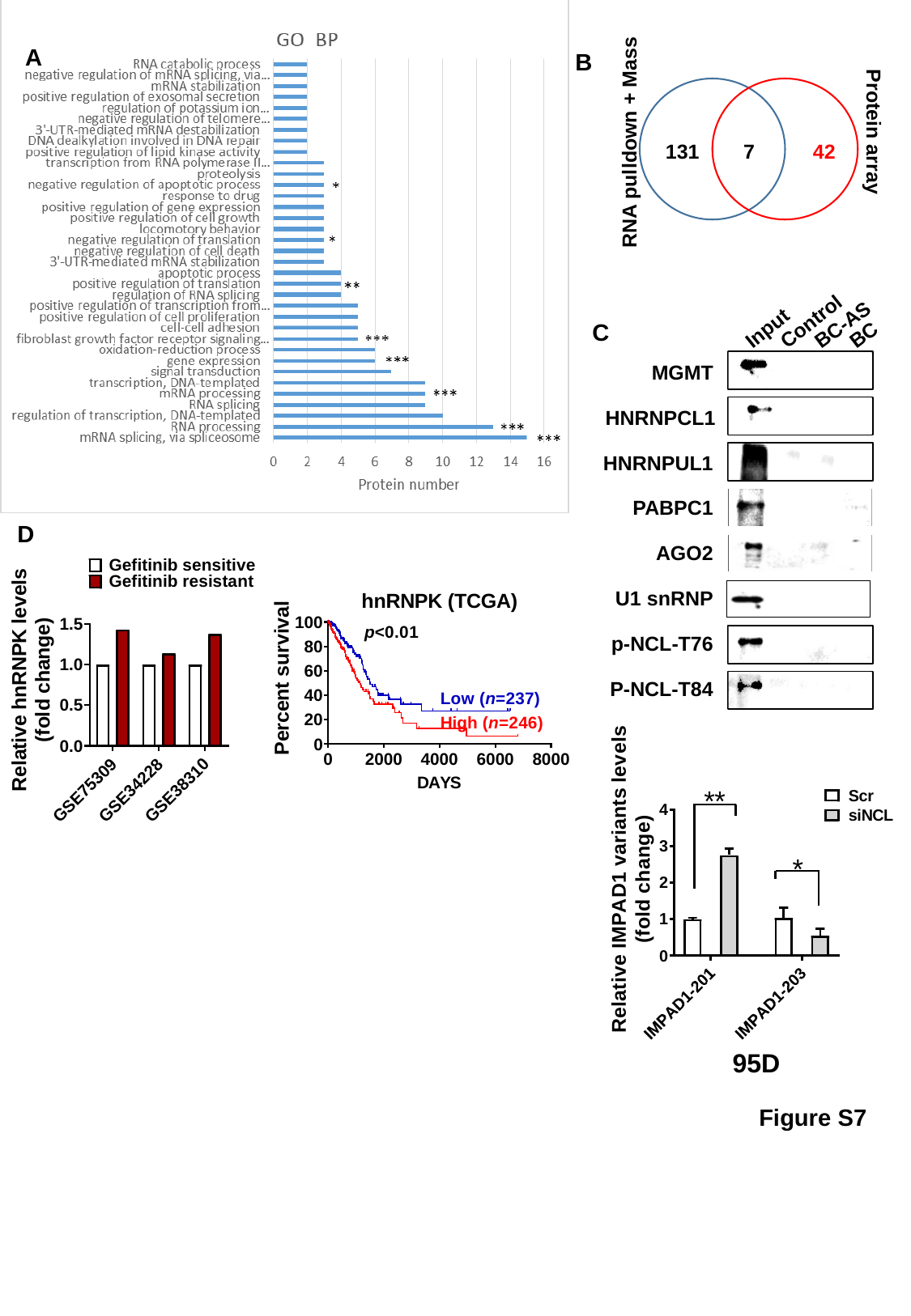

RNA pulldown + Mass
Protein array
131
7
42
A
B
BC-AS
Control
Input
BC
MGMT
HNRNPCL1
HNRNPUL1
PABPC1
AGO2
U1 snRNP
p-NCL-T76
P-NCL-T84
C
D
Figure S7

## Slide 8
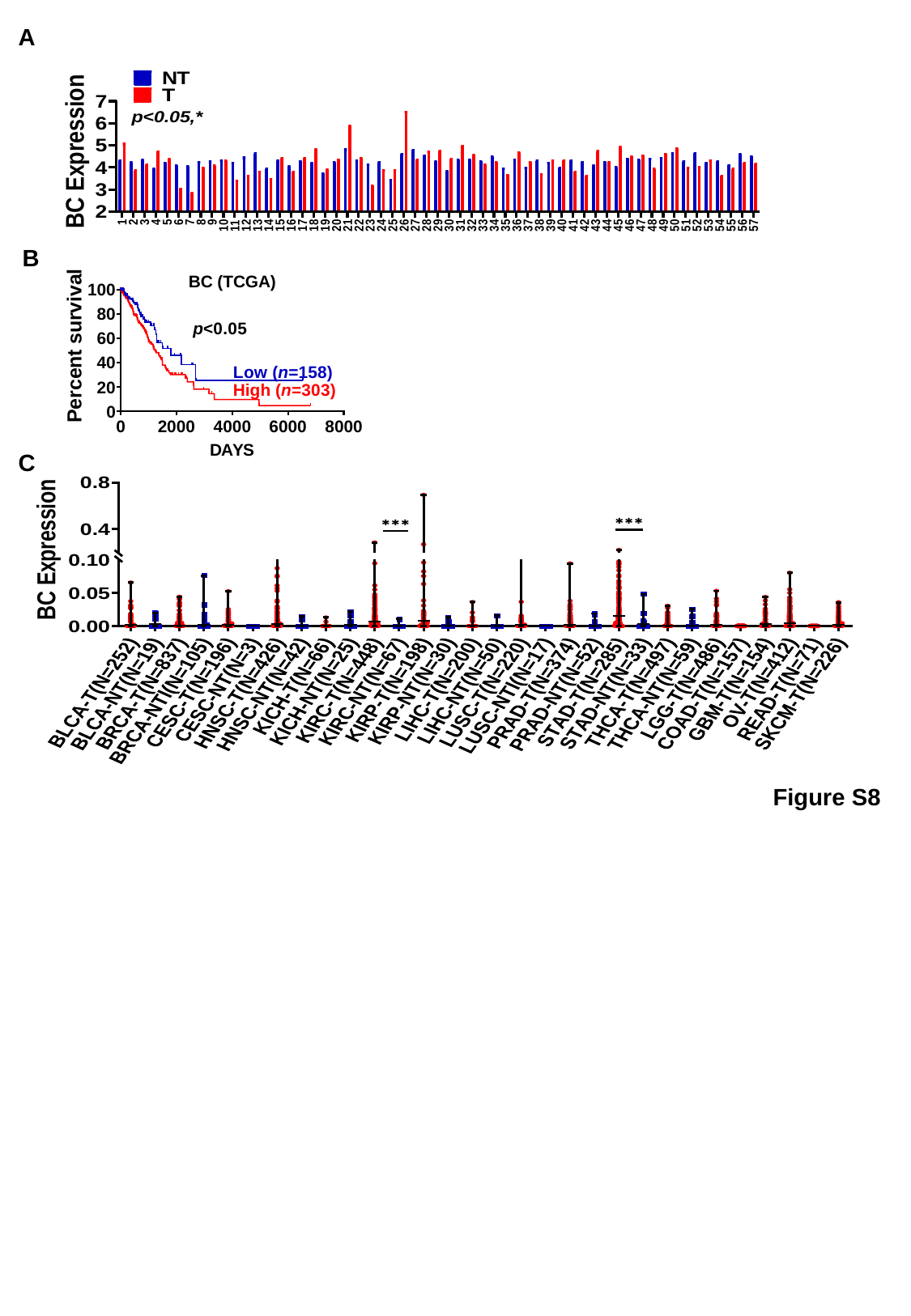

A
B
C
 Figure S8
