# Supplementary material for: LncRNA BC promotes lung adenocarcinoma progression by modulating IMPAD1 alternative splicing
Source: Clin Transl Med. 2023 Jan 17;13(1):e1129. doi: 10.1002/ctm2.1129 (PMC9845120; doi:10.1002/ctm2.1129)
Supplement: Supplementary file 2 — Figure S1 Functional analysis of lncRNA BC Figure S2 BC promoted lung cancer metastasis Figure S3 Domain mapping of lncRNA BC Figure S4 BC enhanced epithelial–mesenchymal transition Figure S5 IMPAD1 association with EMT in lung cancer cells Figure S6 BC regulated IMPAD1 alternative splicing Figure S7 Proteins identified in the BC complex Figure S8 BC expression analysis in LUAD and other tumours Table S1 Summary of primers used in the study Table S2 List of top differential expression LncRNAs (95C vs. 95D, fold change > 3) Table S3 Potential BC binding proteins [file CTM2-13-e1129-s002.docx]

**Figure S1. Functional analysis of lncRNA BC.**

**A.** The coding probability of the BC sequence was analyzed using the Coding Potential Assessment Tool (CPAT) with coding integrin αv subunit (ITGAV), and non-coding lncRNA TINCR, HULC, LOC100132707, AL589182.3 as controls (upper). The conservation of BC009639 across species was analyzed using the Multiz alignments tool of the UCSC Genome Browser (lower).

**B.** According to the expression of BC in LUAD, the heat map and gene list correlation profile of all features in the GO: epidermal growth factor receptor signaling pathway gene set (top). Epidermal growth factor receptor signaling pathway in gene set enrichment analysis based on BC expression from the TCGA database (left) and in LUAD compared with non-tumor (middle), KEGG:_EGFR tyrosine kinase inhibitor resistance in gene set enrichment analysis based on BC expression from the TCGA database (right).

**C.** Gene ontology analysis of BC-related genes according to the TANRIC database.

**Figure S2. BC promoted lung cancer metastasis.**

1. Representative micrographs of transmembrane migration (Magnification: 20×) in 95D cells with BC overexpression or knockout. Quantitative analysis on the right.
2. Representative micrographs of invasion assay (Magnification: 20×) in 95D cells with BC overexpression or knockout. Quantitative analysis on the right.
3. Representative micrographs of wound healing (Magnification: 10×) in 95D cells with BC overexpression or knockout. Quantitative analysis on the right.
4. Phosphorylation of EGFR(Tyr1173) and VEGFR(Tyr951) were measured after overexpressing BC, 10ng/ml EGF or VEGF treatment as control .

Data are represented as mean ± SEM of at least three independent experiments. * *p* <0.05, ** *p* <0.01.

**Figure S3. Domain mapping of lncRNA BC.**

1. Secondary structure of the BC molecule predicted by RNAfold web analysis (upper). Schematic diagram of BC mutations by domain deletion (lower). D, domain.
2. Cell viability assay in cells transfected with BC domain mutants.

**C, D.** Representative micrographs of transmembrane migration (Magnification: 20×) and wound healing of 95D cells with BC domain 2 or domain 3 overexpression.

Data are represented as mean ± SEM of at least three independent experiments. * *p* <0.05, ** *p* <0.01.

**Figure S4. BC enhanced epithelial–mesenchymal transition.**

1. Pearson correlation analysis between BC expression and cadherin, vimentin, Snai1 or ZEB1 expression using data from the TCGA database.
2. The expression of Cadherin, vimentin, Snai1, Slug, ZEB-1, and AKT were determined by Western blot in PC9 cells with BC overexpression (upper). Quantification of western blots in 95C, 95D, A549 and PC9 cells with BC overexpression (lower).
3. HALLMARK:_Epithelial_Mesenchymal_Transition pathway in gene set enrichment analysis based on BC expression from the TCGA database.
4. HALLMARK:_Epithelial_Mesenchymal_Transition pathway in gene set enrichment analysis of BC overexpressing cells RNA-seq data.

**Figure S5. IMPAD1 association with EMT in lung cancer cells.**

1. Representative micrographs of wound healing (upper) (Magnification: 10×) in 95D cells with IMPAD1 overexpression. Quantitative analysis on the right. Representative micrographs of transmembrane migration (lower) (Magnification: 20×) in 95D cells with IMPAD1 overexpression. Quantitative analysis on the right.
2. Viability of IMPAD1-overexpressing PC9 cells was measured by the MTT assay after treatment with various concentrations of gefitinib, afatinib, or osimertinib for 24 h. The IC50 of the EGFR-TKIs was also measured.
3. IMPAD1, Cadherin, vimentin, Snai1, ZEB-1, Slug, and AKT were detected by Western analysis in A549 cells overexpressing IMPAD1.
4. Phosphorylation of AKT, PDPK1, Src, and mTOR in A549 cells were measured after overexpressing IMPAD1.
5. IMPAD1, E-cadherin, Slug, Src, PDPK1, and phosphorylated Src and PDPK1 were determined by Western blot in A549 cells transfected with the BC D2 mutant.

Data are represented as mean ± SEM of at least three independent experiments. * *p* <0.05, ** *p* <0.01.

**Figure S6. BC regulated IMPAD1 alternative splicing.**

1. Ideograph of IMPAD1-201 and IMPAD1-203 transcript variants (upper). Ideograph of IMPAD1-203 exon 3’ splicing (lower).
2. Statistics of alternative splicing events in BC overexpressing lung cancer cells based on RNA-Seq data. A5SS, Alternative 5'splice site. A3SS, Alternative 3'splice site. SE, Skipped exon. RI, Retained intron. MXE, Mutually exclusive exons.
3. IMPAD1 transcript variants in BC overexpression lung cancer cells visualization with IGV (Vision IGV_2.10.2)

**Figure S7. Proteins identified in the BC complex.**

**A.** Gene ontology analysis of potential BC-binding proteins from protein microarray results. * *p* <0.05, ** *p* <0.01, *** *p* <0.001

**B.** The numbers in the circles represent the binding proteins identified by mass spectrometry (blue) and protein microarray (red). The overlapped part represents the proteins common to both mass spectrometry and protein microarray.

**C.** Binding of BC to P-NCL, MGMT, PABPC1, AGO2, and U1 snRNP was analyzed by BC RNA pull-down assay (upper). The IMPAD1-201 and IMPAD1-203 splice variants were detected by RT-qPCR in 95D cells after NCL knockdown.

**D.** Expression of hnRNPK was measured in gefitinib-sensitive or -resistant lung cancer cells from public dataset GSE75309, GSE34228, and GSE38310 (left). Kaplan–Meier analysis of overall survival of 483 patients with LUAD based on hnRNPK expression from the TCGA data.

**Figure S8. BC expression analysis in LUAD and other tumors.**

1. BC expression analysis of 57 pairs of LUAD tissues (T) and adjacent non-tumor (NT) lung tissues from the TCGA database.
2. Kaplan–Meier analysis of overall survival of 461 patients with LUAD based on BC expression from the TCGA database.
3. BC expression analysis in various tumor and non-tumor tissues from the TCGA database. BLCA, bladder urothelial carcinoma; BRCA, breast invasive carcinoma; CESC, cervical squamous cell carcinoma and endocervical adenocarcinoma; HNSC, head and neck squamous cell carcinoma; KIRC, kidney renal clear cell carcinoma; KIRP, kidney renal papillary cell carcinoma; LIHC, liver hepatocellular carcinoma; LUSC, lung squamous cell carcinoma; PRAD, prostate adenocarcinoma; STAD, stomach adenocarcinoma; THCA, thyroid carcinoma; UCEC, uterine corpus endometrioid carcinoma; LGG, brain lower grade glioma; COAD, colon adenocarcinoma; GBM, glioblastoma multiforme; OV, ovarian serous cystadenocarcinoma; READ, rectum adenocarcinoma; SKCM, skin cutaneous melanoma.

Supplemental Table 1. Summary of primers used in the study

|  | Primers for PCR (5'-3') |
| --- | --- |
| GAPDH F | CGGATTTGGTCGTATTGGG |
| GAPDH R | CGCTCCTGGAAGATGGTGAT |
| BC009639 F | AGGTCATAACACGCTAAG |
| BC009639 R | AACATAGATTGAAAACT |
| IMPAD1F641 | TACCTGCTCAAGACCG |
| IMPAD1R1135 | ACTCTTATCAGGCACATC |
| IMPAD203S227 | TATCTTGCCCTGTAGTGAGT |
| IMPAD1-203-E3-F | AGGATCTTCGAAAGTACGTCA |
| IMPAD1-203-E3-AS | TCTACCATTGCCCAAGTCA |
| HNRNPK-F813 | CTTTGACTGCGAGTTGAGGC |
| HNRNPK-R922 | TGATGGTGGTTTGAGTGTTCT |
|  | shRNA (5'-3') |
| shHNRNPK S | CUGCUCUGCAAGGAUAUAUUU |
| shHNRNPK AS | AUAUAUCCUUGCAGAGCAGUU |
| shIMPAD1 S | GGUAGAUGGUGGUUCAAAUUU |
| shIMPAD1 AS | AUUUGAACCACCAUCUACCUU |
|  | siRNA (5'-3') |
| si-NCL | GGGCAAAGAAUGGCAAGAAUUUUCUUGCCAUUCUUUGCCCUU |
|  | gRNA or Primers for CRISPR-Cas9 system (5'-3') |
| gRNA-F | 5’GACTATCATATGCTTACCGTAACT3’ |
| gRNA-R | 5’CAAGTTGATAACGGACTAGCCTTA3’ |
| g5-F | 5’ACACCTTGGTCATGTCTAAAAGAGAGTTTTAGAGCTAGAAATAGCAAGTTAAAATAAGGCTAGTCCGTT3’ |
| g5-R | 5’AACGGACTAGCCTTATTTTAACTTGCTATTTCTAGCTCTAAAACTCTCTTTTAGACATGACCAAGGTGT3’ |
| g265-F | 5’ACACCGGGCACAGGGATTTTAGCAGGTTTTAGAGCTAGAAATAGCAAGTTAAAATAAGGCTAGTCCGTT3’ |
| g265-R | 5’AACGGACTAGCCTTATTTTAACTTGCTATTTCTAGCTCTAAAACCTGCTAAAATCCCTGTGCCCGGTGT3’ |
|  | Primers for plasmid construction (5'-3') |
| BC009639F1D1 | TTTTTTTTTTTTTTTAGACATGA |
| BC009639R123D1 | GCTGTAGATAATGTGATTGCTTC |
| BC009639F124D2 | AAATTGCCTGGGGTAGTATCT |
| BC009639R330D2 | CCCAGTCTCTTGCACAAATTA |
| BC009639F331D3 | CTATGTGCTGGGGGTGAG |
| BC009639R719D3 | CTATGTTCTTGCCTCTTCATAC |
| BC009639F720D4 | ATTGAAAACTCCATTTCCTAGT |
| BC009639R765D4 | ACAAGGGATTGAGTTTACACTAA |
| IMPAD1 F287 BamHⅠ | AAAGGATCCATGGCCCCCATGGGC |
| IMPAD1 R1363 XhoⅠ | CCCCCTCGAGTTTATGTCCTGTCTTTTCTAGATCT |

Supplemental Table 2. List of top differential expression LncRNAs (95C vs. 95D, fold change>3).

| LncRNAs | 95C | 95D |
| --- | --- | --- |
| chr20:53108150-53174325 | 56.136894 | 3923.4062 |
| chr2:106659950-106724850 | 303.5143 | 18395.127 |
| HMlincRNA135 | 123.92102 | 5241.4526 |
| HMlincRNA915 | 53.2712 | 2136.46 |
| uc.360- | 71.79659 | 2715.4521 |
| HMlincRNA114 | 232.79672 | 8222.952 |
| chr2:63825225-63857575 | 57.4496 | 1899.3998 |
| chr1:110140000-110161150 | 76.47579 | 2261.795 |
| HMlincRNA1114 | 51.213898 | 1323.5144 |
| chr5:153455300-153511350 | 56.136894 | 1111.679 |
| HMlincRNA1577 | 62.5864 | 1232.7943 |
| chr13:48083800-48168625 | 51.865196 | 909.0144 |
| HMlincRNA1480 | 51.865196 | 823.40436 |
| chr8:140183025-140239350 | 115.10803 | 1815.6244 |
| uc001flf | 208.41913 | 3214.7944 |
| uc002ret | 84.697685 | 1267.959 |
| chr4:84354450-84393075 | 99.572586 | 1481.9591 |
| chrX:112699025-113202875 | 64.9645 | 888.6246 |
| chr2:40800500-41088800 | 50.553596 | 669.28925 |
| AK128498 | 76.77941 | 1005.3992 |
| chr2:67455100-67475300 | 754.9044 | 9850.236 |
| HMlincRNA1448 | 124.69479 | 1605.68 |
| HMlincRNA1321 | 57.8462 | 731.0694 |
| HMlincRNA111 | 56.662796 | 709.2343 |
| chr10:53930025-54182600 | 97.91553 | 1218.2393 |
| chrX:113383525-113403350 | 70.14661 | 789.1794 |
| HMlincRNA189 | 62.74271 | 686.90436 |
| chr12:95477300-95516900 | 62.058887 | 658.06934 |
| chr14:52757725-52912850 | 63.844208 | 676.17944 |
| chrX:106786484-106830375 | 89.152794 | 943.67914 |
| chr2:143157200-143262500 | 57.8462 | 604.39923 |
| chr10:14583425-14595550 | 275.78934 | 2731.1804 |
| chr1:41622050-41638350 | 58.53938 | 578.6243 |
| chr14:95159375-95182150 | 57.055904 | 552.84424 |
| M91159 | 51.865196 | 497.8993 |
| chr3:109306850-109354500 | 57.8462 | 554.349 |
| chr1:9129450-9164075 | 106.72962 | 1016.899 |
| chr3:72727375-72817150 | 51.1649 | 479.28955 |
| chr2:28505600-28531825 | 57.564384 | 533.8993 |
| chr5:141603100-141658125 | 78.58472 | 726.79443 |
| chr1:171117525-171191468 | 85.02821 | 782.5145 |
| chr2:134595725-134694600 | 58.53938 | 537.79425 |
| chr6:107039875-107055450 | 136.0907 | 1219.1294 |
| HMlincRNA583 | 111.49532 | 989.5693 |
| HMlincRNA1074 | 54.93759 | 466.45926 |
| chr8:114557150-115165825 | 56.662796 | 478.39948 |
| chr4:84354450-84393075 | 73.502815 | 615.62427 |
| chr6:119217000-119228400 | 51.316494 | 424.95938 |
| HMlincRNA1058 | 196.10342 | 1608.0146 |
| chr1:41622050-41638350 | 59.032993 | 478.2341 |
| HIV1884 | 53.09791 | 429.6116 |
| chr13:72807725-72937000 | 57.0003 | 457.34412 |
| uc010gxm | 52.1177 | 406.05066 |
| chr16:85445825-85562825 | 102.1768 | 795.0144 |
| chr10:31608475-31625250 | 53.2712 | 411.51416 |
| HMlincRNA1127 | 132.87471 | 1014.5691 |
| chr5:54056975-54069450 | 56.552517 | 431.40417 |
| uc003wmh | 147.26779 | 1118.7894 |
| chr6:126536000-126549075 | 50.9207 | 380.62927 |
| HMlincRNA1074 | 57.3909 | 427.34427 |
| HMlincRNA1513 | 56.884106 | 406.84433 |
| chr9:117609800-117640825 | 59.439186 | 420.2942 |
| chr16:73375750-73388725 | 60.99011 | 430.39926 |
| chr5:176041950-176075125 | 50.5053 | 351.9593 |
| uc002ulj | 66.86731 | 462.6407 |
| HMlincRNA199 | 51.2674 | 354.01428 |
| HMlincRNA1153 | 96.960686 | 667.23425 |
| HMlincRNA460 | 65.355804 | 448.0693 |
| HMlincRNA819 | 58.797005 | 402.50943 |
| HMlincRNA1137 | 221.68501 | 1517.07 |
| chr5:153455300-153511350 | 50.141888 | 342.12433 |
| chr3:131414550-131449200 | 79.71091 | 533.5691 |
| HMlincRNA76 | 103.2595 | 690.789 |
| chr3:36894500-36969325 | 82.45613 | 547.5694 |
| HMlincRNA1074 | 109.879105 | 728.0143 |
| chr2:114283350-114336075 | 140.39822 | 925.0141 |
| HMlincRNA732 | 62.74271 | 407.5692 |
| chr12:71550025-71815050 | 71.48609 | 461.68445 |
| chr1:65291650-65304400 | 50.698605 | 324.78943 |
| HMlincRNA63 | 82.093506 | 521.56934 |
| chr3:156892700-156920200 | 125.62319 | 792.45966 |
| chr2:118641200-118655550 | 73.4305 | 456.84918 |
| uc004eyh | 59.322784 | 362.67917 |
| HMlincRNA117 | 73.13899 | 446.90442 |
| chr2:19091050-19143450 | 164.854 | 1003.6793 |
| chr2:237685400-237732944 | 52.780003 | 320.1794 |
| chr14:68342850-68361800 | 351.40436 | 2117.794 |
| HMlincRNA1153 | 61.050007 | 364.79434 |
| chr9:109835775-109863175 | 77.23827 | 460.34933 |
| HMlincRNA1004 | 56.717995 | 334.2942 |
| chr16:47237250-47265300 | 50.141888 | 294.79425 |
| AK024824 | 51.765694 | 302.67923 |
| uc003naa | 51.4228 | 298.6123 |
| chr4:116354750-116650400 | 100.4514 | 582.1794 |
| chrX:57186025-57301200 | 56.136894 | 323.89923 |
| HMlincRNA120 | 56.218 | 323.89923 |
| NR_024418 | 51.7 | 297.40356 |
| chr9:136571925-136590625 | 55.668896 | 319.17923 |
| HMlincRNA1530 | 51.36569 | 293.6293 |
| HMlincRNA595 | 232.57185 | 1316.6791 |
| chr7:37994575-38045650 | 56.082294 | 317.45926 |
| HMlincRNA1584 | 91.354195 | 513.84924 |
| chr15:72297100-72307675 | 68.18072 | 375.0144 |
| chr7:137497281-137771725 | 242.7354 | 1334.8444 |
| chrX:67684350-67726050 | 63.653015 | 349.95416 |
| chr8:91182275-91466575 | 64.609604 | 354.7942 |
| NR_024491 | 69.702286 | 382.19827 |
| chr1:27717600-27729175 | 110.61301 | 606.124 |
| chr18:31720150-31782325 | 79.6333 | 436.2391 |
| uc002wsk | 50.099796 | 272.91522 |
| uc.227+ | 62.89409 | 340.43887 |
| chr1:185637825-185716750 | 63.306786 | 341.50928 |
| uc010enn | 52.471405 | 279.9043 |
| chr1:89521625-89559825 | 76.17338 | 406.23914 |
| ASO1977 | 180.69385 | 963.58344 |
| HMlincRNA911 | 79.24288 | 422.12436 |
| chr2:127007450-127034900 | 88.68389 | 468.23935 |
| chr10:116533050-116571161 | 73.06392 | 385.5692 |
| chrX:78326100-78453100 | 51.865196 | 270.17944 |
| chr2:191398300-191442800 | 60.8106 | 315.01428 |
| DQ372724 | 66.567406 | 342.54684 |
| HMlincRNA133 | 88.937096 | 456.6496 |
| chr5:102017350-102033375 | 54.775497 | 280.51428 |
| HMlincRNA460 | 78.0802 | 398.8443 |
| uc003wts | 110.92709 | 566.43524 |
| uc004fnm | 92.011314 | 468.84454 |
| HMlincRNA879 | 72.84861 | 370.34433 |
| AK022108 | 82.5325 | 417.15457 |
| chr3:111003125-111018900 | 126.39581 | 633.8492 |
| BC009639 | 80.387794 | 396.44598 |
| chr3:152670000-152720900 | 53698.777 | 58.419697 |
| chr17:10598925-10629325 | 31600.89 | 70.953 |
| chr13:102930525-103035800 | 7174.513 | 111.986786 |
| chrX:78326100-78453100 | 6085.6255 | 158.61305 |
| chr2:204328625-204374375 | 1171.7394 | 50.969414 |
| chr17:45290908-45308137 | 1107.6842 | 52.881805 |
| chr11:76004575-76019350 | 6788.347 | 325.0693 |
| chr4:84354450-84393075 | 3081.5151 | 158.0727 |
| chr6:57029975-57042500 | 1495.7949 | 86.94493 |
| chr8:118825700-118840650 | 1437.069 | 103.53977 |
| chr8:101561650-101582050 | 887.9592 | 70.4237 |
| chr12:4208850-4236425 | 970.6246 | 77.3895 |
| HMlincRNA742 | 663.39923 | 56.218 |
| chr2:146013750-146223325 | 760.569 | 73.286285 |
| chr10:118564075-118598450 | 784.6242 | 76.24809 |
| HMlincRNA854 | 584.4594 | 57.111603 |
| chrX:108881550-109095625 | 532.899 | 52.3706 |
| CR617813 | 4949.907 | 494.01434 |
| chr8:8935475-8948675 | 565.17926 | 58.912205 |
| HMlincRNA239 | 624.4593 | 68.04632 |
| chr14:65797475-66001975 | 1049.6844 | 115.25589 |
| HMlincRNA339 | 783.9596 | 86.60873 |
| chr11:129971550-129983400 | 605.4045 | 69.5545 |
| chr15:46800350-46810550 | 622.5695 | 72.199875 |
| chr18:35328475-35629325 | 3804.796 | 446.06915 |
| uc.343+ | 503.2858 | 60.371803 |
| NR_024248 | 559.5693 | 67.539185 |
| uc010iuf | 1202.1128 | 148.3524 |
| chr20:55589775-55605050 | 412.29425 | 51.01819 |
| chr8:114557150-115165825 | 896.8492 | 111.15101 |
| HMlincRNA249 | 1081.4595 | 136.1432 |
| chr5:85778600-85902825 | 568.9593 | 74.381195 |
| chr11:44677300-44698225 | 436.62424 | 57.33491 |
| chr2:127007450-127034900 | 847.6789 | 114.55652 |
| chr12:32445125-32538825 | 1791.2942 | 242.4054 |
| HMlincRNA897 | 439.51453 | 59.561195 |
| HMlincRNA1017 | 469.06927 | 64.13029 |
| uc002yjk | 401.5092 | 56.828705 |
| HMlincRNA1089 | 499.5143 | 70.883194 |
| HMlincRNA1592 | 354.51425 | 50.871998 |
| AK022220 | 462.4823 | 66.455185 |
| HMlincRNA697 | 924.6296 | 133.8748 |
| CR612530 | 385.7974 | 57.95881 |
| uc001vlb | 459.18442 | 69.623085 |
| chr1:51794250-51821775 | 821.6791 | 126.86667 |
| HMlincRNA460 | 352.1243 | 54.616394 |
| chr2:127007450-127034900 | 558.9592 | 87.960106 |
| chr1:35058475-35091575 | 332.06927 | 52.269905 |
| HMlincRNA1177 | 315.8494 | 50.237606 |
| uc003wbg | 406.06946 | 64.9645 |
| chr2:16335875-16523025 | 319.17923 | 51.36569 |
| chrX:68155600-68165750 | 665.6241 | 107.35747 |
| chr16:82518800-82529525 | 353.23422 | 57.4496 |
| chr20:53108150-53174325 | 445.3494 | 72.9203 |
| NR_001296 | 1186.6792 | 194.38759 |
| chr8:6541450-6552025 | 11718.509 | 1949.3993 |
| HMlincRNA1338 | 912.1245 | 151.80948 |
| chrX:62428575-62446375 | 544.6241 | 92.54059 |
| CR593590 | 330.96042 | 56.269993 |
| HMlincRNA1576 | 1268.9039 | 217.13225 |
| chrX:51150500-51160675 | 295.3443 | 50.823395 |
| chr8:114557150-115165825 | 2270.0151 | 391.0693 |
| uc010iyf | 692.3291 | 119.75579 |
| chr17:41006725-41017400 | 514.2893 | 89.53928 |
| NR_027028 | 958.7838 | 167.23141 |
| uc004aut | 1006.34467 | 175.57812 |
| uc010avf | 494.67932 | 86.778496 |
| chr4:113827100-114125350 | 300.29428 | 52.8309 |
| HMlincRNA583 | 324.12433 | 57.055904 |
| chr7:7895500-7963275 | 323.56924 | 57.564384 |
| HMlincRNA734 | 781.01434 | 139.86499 |
| HMlincRNA1547 | 340.56943 | 61.235786 |
| BC020163 | 628.1809 | 113.336525 |
| chr2:19091050-19143450 | 379.95932 | 68.93371 |
| chr20:48468950-48485200 | 482.00928 | 87.4951 |
| chr8:129364550-129425050 | 289.18423 | 52.6784 |
| HMlincRNA1539 | 322.89917 | 59.090813 |
| chr20:57146000-57188600 | 355.84927 | 65.355804 |
| L08437 | 283.95435 | 53.142 |
| chr2:85975525-86007075 | 344.12442 | 64.41882 |
| HMlincRNA360 | 310.67914 | 58.479603 |
| uc003wax | 266.62445 | 50.18971 |
| HMlincRNA1074 | 609.0145 | 114.708984 |
| chrX:62428575-62446375 | 899.62445 | 171.9476 |
| chr7:30183275-30279675 | 436.84424 | 84.0399 |
| uc001vbt | 282.56927 | 54.56349 |
| HMlincRNA1377 | 514.4542 | 99.4771 |
| AK129631 | 256.0147 | 50.0941 |
| HMlincRNA254 | 278.9595 | 54.85431 |
| HMlincRNA1378 | 258.7897 | 51.01819 |
| BC073933 | 797.5673 | 157.41457 |
| uc003tbl | 389.45926 | 77.15928 |
| uc003jxm | 1116.7338 | 221.575 |
| uc010iyc | 1754.4047 | 349.29434 |
| AK123491 | 510.89987 | 101.81952 |
| uc001zeg | 403.42282 | 80.594 |
| NR_024477 | 468.40402 | 93.90938 |
| HMlincRNA711 | 309.4592 | 62.463295 |
| HMlincRNA111 | 249.34499 | 50.6502 |
| uc010gdr | 371.34427 | 75.79769 |
| chr8:124540800-124568875 | 398.0094 | 81.85151 |
| uc002ybr | 858.38696 | 177.33495 |
| uc004cni | 243.19675 | 50.4637 |
| uc003wbn | 361.23425 | 75.6459 |
| HMlincRNA268 | 491.18433 | 102.975494 |

Supplemental Table 3. Potential BC binding proteins.

| Protein array | RNA pulldown + Mass |
| --- | --- |
| HNRNPK | HRNR |
| NIPAL3 | VIM |
| TIA1 | LMNA |
| RBM3 | HSPA5 |
| MGMT | DSP |
| HNRNPD | DSG1 |
| PABPC1 | HSPA8 |
| HNRNPF | PHB |
| PTBP3 | HYOU1 |
| HNRNPC | HSPA9 |
| SMPD1 | PDIA4 |
| PABPC5 | NPM1 |
| HNRNPAB | HIST1H4A |
| CSTF2 | ALB |
| PCBP3 | HSPD1 |
| SF3B4 | TUBB |
| NOVA1 | NCL |
| RALYL | ANXA2 |
| MSI2 | TUBA1A |
| YBX3 | ATP5A1 |
| RBM38 | TUBB2A |
| MPG | DCD |
| CPNE2 | H1F0 |
| IGFBP1 | CSTA |
| YBX2 | ACTB |
| WISP2 | HSPA1L |
| TFAP2E | EEF1A1 |
| ACOT7 | HIST1H1C |
| POLB | HNRNPU |
| QKI | SYNCRIP |
| CPT1A | RPL7 |
| CPEB4 | HIST1H2AB |
| NCL | SLC25A5 |
| ESRP1 | SLC25A6 |
| SLC35E3 | RPL6 |
| NAMPT | HNRNPR |
| RBPMS | RPL8 |
| HNRNPA1 | HIST1H1B |
| CRYZ | RPL18 |
| SSBP1 | HSP90B1 |
| BDH2 | MATR3 |
| MEX3C | SBSN |
| U2AF2 | GAPDH |
| MARCKSL1 | PARP1 |
| Hnrnph1 | CDCA8 |
| XAGE1A | NOLC1 |
| N4BP1 | ATP5B |
| SH3BP1 | HNRNPM |
| IGF2BP1 | DES |
|  | JUP |
|  | DSC1 |
|  | IGF2BP1 |
|  | FLG2 |
|  | DLAT |
|  | RPN1 |
|  | RPN2 |
|  | TAF15 |
|  | RPS3 |
|  | HNRNPK |
|  | RPLP1 |
|  | RPS4X |
|  | FUS |
|  | DDX5 |
|  | IWS1 |
|  | HIST1H2BB |
|  | SLC25A11 |
|  | RPL4 |
|  | CALML5 |
|  | PSMA8 |
|  | RPS23 |
|  | RPL29 |
|  | YWHAZ |
|  | PKP1 |
|  | LYZ |
|  | HSP90AB1 |
|  | HIST1H1T |
|  | PIP |
|  | NEFL |
|  | HSPA6 |
|  | ATP5C1 |
|  | PKM |
|  | EEF2 |
|  | HNRNPH1 |
|  | RPLP0 |
|  | G3BP1 |
|  | LMNB2 |
|  | TRAP1 |
|  | HNRNPD |
|  | UBB |
|  | SLC3A2 |
|  | S100A7 |
|  | BLMH |
|  | AIFM1 |
|  | S100A9 |
|  | TXN |
|  | RPL7A |
|  | INA |
|  | PHB2 |
|  | AZGP1 |
|  | TGM1 |
|  | RPL11 |
|  | LRP5 |
|  | LMNB1 |
|  | XRCC6 |
|  | NOP2 |
|  | CDSN |
|  | ARG1 |
|  | CTSD |
|  | TPI1 |
|  | RPS14 |
|  | HNRNPCL1 |
|  | RPS2 |
|  | GNB1 |
|  | PRDX1 |
|  | VDAC2 |
|  | VDAC1 |
|  | VDAC3 |
|  | GCN1L1 |
|  | DDX3Y |
|  | SFPQ |
|  | CASP14 |
|  | SNRPB |
|  | SERPINB12 |
|  | TMPRSS11E |
|  | PABPC1 |
|  | PGK1 |
|  | SRSF3 |
|  | LDHA |
|  | SNRPA1 |
|  | S100A8 |
|  | RPS16 |
|  | HBB |
|  | SMC6 |
|  | RPS25 |
|  | HNRNPL |
|  | ERI2 |
|  | SF3B4 |
|  | RBM3 |

**BC009639 sequence:**

>BC009639.2 Homo sapiens cDNA clone IMAGE:3897094, partial cds

TTTTTTTTTTTTTTTAGACATGACCAATTTATTCAGAGAATTCAAATTTCGTTTGGCAAAGTATATCCGGGGCAGAGAGTTTGGGATAATTATGTCATTGGAAGCAATCACATTATCTACAGCAAATTGCCTGGGGTAGTATCTGAAGGAAAGGCAAAACTTTTAAAAACAATTTAGTATGTGGGGGGGTGATAATCATAAATATTTGCAAAGGTAACAAAACAAACAACCAGCTTATACAACCAAGGCACAAAATATGCTAATGCTAATAATCCTTTATTCAATTTAGCTCAACACACATTAAGTACTTAATTTGTGCAAGAGACTGGGCTATGTGCTGGGGGTGAGGTGGAAATACAAAAACACCAAGATGCAATCCCTCTCAAGAACTGTATAATCTAGTAAGAGCACATACAGAGATGGTGCTTGCAGGTAAAAACTGCTCTGAAACCATGGGGAGAGAAGAGTTTACTTCCTTCCAGAGGGTGAAGTCGGGACCCATTTAAATTTGGTAGTATGGGTGAGGAAGGTCATAACACGCTAAGTAAACTGGTGTCTAAGCATGTGACGGCAACAGCTAATGGTCTAGTTCCTCCATGGCTTTAAATGCATGAAAGGGAAAAGAGTATTCAAAGGTATTTTTATTTTATCTCATTGTTAGCCCAGTATAAGGCAGGATGACAAAAAATAAATAAAAGTATGAAGAGGCAAGAACATAGATTGAAAACTCCATTTCCTAGTTTTAGTGTAAACTCAATCCCTTGTGCATATACATCTAGTTCCTGAAGTCCACACTGCCAAAAGGGAAAAACAAGAAAAACCAGCCCTAGCAGTGCCCTGTCATCATGGCAGAGCACTGTCTCTTCTGTGGGACGACTAGTTCTAGACCGCGA
